# Analysis of Human Endogenous Retrovirus Expression in Multiple Sclerosis Plaques

**DOI:** 10.16966/2473-1846.133

**Published:** 2017-07-24

**Authors:** PJ Bhetariya, JD Kriesel, KF Fischer

**Affiliations:** 1 Department of Internal Medicine, Division of Infectious Diseases, University of Utah School of Medicine, Salt Lake City, Utah, USA; 2 Department of Pathology, University of Utah School of Medicine, Salt Lake City, Utah, USA

**Keywords:** Human endogenous retroviruses, HERV, qPCR, Multiple sclerosis, Primary progressive multiple sclerosis, GAG, Envelope, Gene expression

## Abstract

**Background:**

It has been suggested that Human endogenous retroviruses (HERVs) are associated with multiple sclerosis (MS) pathogenesis. The objective of this study was to broadly evaluate the expression of HERV core (GAG) and envelope (ENV) genes in diseased brain white matter samples from MS patients compared to normal controls.

**Methods:**

Twenty-eight HERV GAG and 88 ENV gene sequences were retrieved, classified by phylogeny, and grouped into clades. Consensus qPCR primers were designed for each clade, and quantitative PCR was performed on 33 MS and 9 normal control frozen brain samples. MS samples included chronic progressive (n=5), primary progressive (n=4), secondary progressive (n=14), relapsing remitting (n=3) and unclassified confirmed MS cases (n=7). The levels of GAG and ENV RNA within each of the samples were quantitated and normalized using the neuronal reference gene RPL19. Expression differences were analyzed for MS *vs* control.

**Results:**

Expression of GAG clades 1A, 3B, and 3C mapping to HERV-E and HERV-K were significantly increased compared to controls, while GAG clade 3A expression was decreased. Expression of HERV ENV clades 2, 3A, 3B, mapping to RTVL, HERV-E and HERV-K and MSRV (HERV-W), were significantly increased in the MS group. However, the relative expression differences between the MS and control groups were small, differing less than 1.5-fold.

**Conclusion:**

Expression of GAG and ENV mapping to HERV-E, RTVL and HERV-K10 families were significantly increased in the MS group. However, the relative expression differences between the MS and control groups were small, differing less than 1.5-fold. These results indicate that the expression of HERV GAG and ENV regions do not differ greatly between MS and controls in these frozen brain samples.

## Introduction

Multiple sclerosis (MS) is an autoimmune disease characterized by multiple lesions with plaques in the brain and spinal cord. The disease is associated with an inflammatory process that attacks and destroys the myelin sheaths around axons in the brain and spinal cord. The cause of MS is unknown, although the disease is thought to be triggered by environmental factors operating on a predisposing genetic background. Genome-wide association studies (GWAS) have identified numerous susceptibility loci, mainly associated with immunological processes [1]. The human leukocyte antigen (HLA) immune gene-cluster, particularly HLA-DRB1*1501, is the main genetic risk factor for the disease [2,3].

There has been much speculation that an infectious agent may contribute to MS. Based on the epidemiology of MS, including geographic patterns, isolated outbreaks and migration studies, viruses have long been suspected as causative agents [4–6]. There is no direct evidence for the involvement of an exogenous retrovirus in MS, but endogenous retroviruses may be an important factor in MS pathogenesis. The possibility that retroviruses play a role in the pathogenesis of MS has been considered since the 1980s, when Perron showed reverse transcriptase (RT) activity and retroviral particles in leptomeningeal cells taken from a patient with MS [7–10]. Since that time, HERVs have been detected in blood, CSF, and brain tissue from MS patients, as reviewed by Douville RN, Nath A [11]. Human endogenous retroviruses (HERVs) are parts of the human genome, with approximately 98,000 ERV elements and fragments [12–14]. It is believed that HERVs are remnants of prehistoric exogenous retroviruses, integrated in the human genome during evolution through germ line infection [15]. Several transcripts and proteins of genes from the HERV-W family have been detected in the central nervous system and they have been associated with neuroinflammation [16–18]. Antibodies reactive with GAG or ENV antigens from HERVs can be detected in human sera with elevated levels in patients with autoimmune disease [19].

Previously we performed RNA-seq on frozen human brain specimens and showed statistically significant expression differences between progressive MS and normal controls for some HERV domains [20,21]. Importantly, detection of this signal required using a bioinformatics approach called “comprehensive mapping” which counts all detectable alignments to a given sequencing read, rather than only the most specific match. Furthermore, aggregating sequencing hits by domains suggested that the expression of GAG (core) and ENV (envelope) domains is associated with the MS disease process. The objective of this study was to quantify the expression of selected HERV GAG and ENV domains in additional frozen, cryo preserved brain samples from MS patients in comparison to normal controls. Here, several qPCR experiments were performed to gain an understanding of the overall expression of HERV GAG and ENV genes, divided into multiple HERV clades.

## Materials and Methods

### a. Brain specimens

Cryo preserved white matter from MS plaques (N=33) and normal white matter control brain specimens from patients without any brain diseases (N=9) were obtained from the Rocky Mountain MS Center Tissue Bank (Westminster, CO, USA) and the UCLA Human Brain and Spinal Fluid Resource Center (Los Angeles, CA, USA). The MS samples included; chronic progressive MS (CPMS, n=5), primary progressive MS (PPMS, n=4), secondary progressive (SPMS, n=14), relapsing remitting MS (RRMS, n=3). Confirmed MS cases with no clinical subtype (unclassified MS, n=7) were also included in the study for a total of 33 MS samples. These specimens, as well as accompanying clinical and pathologic information (Table 1), were de-identified before shipment. The research plan was submitted to the University of Utah Health Sciences Center IRB for review. Since this research involved only de-identified post-mortem material, it was found to be exempt from IRB review and oversight (IRB #00028658).

### b. HERV database and Primer design

The retroviral gene catalog (RVGC) was compiled by aligning the protein domains in the Gypsy 2.0 database with the human genome (NCBI build 37.p13) [22]. The RVGC contains human genomic sequences with detectable protein homology (BLASTX) to entries in GyDB 2.0 domains database: core (GAG), envelope (ENV), reverse transcriptase (RT), integrase (INT) SCAN-A2, KRAB-A, dUTPase, RNase H, protease (AP) and chromo domain (CHR) (Supplementary Data). RVCG entries were named with arbitrary unique codes of the form: <domain type>_U<sequential_integer> (Table S1).

**Figure 1:**
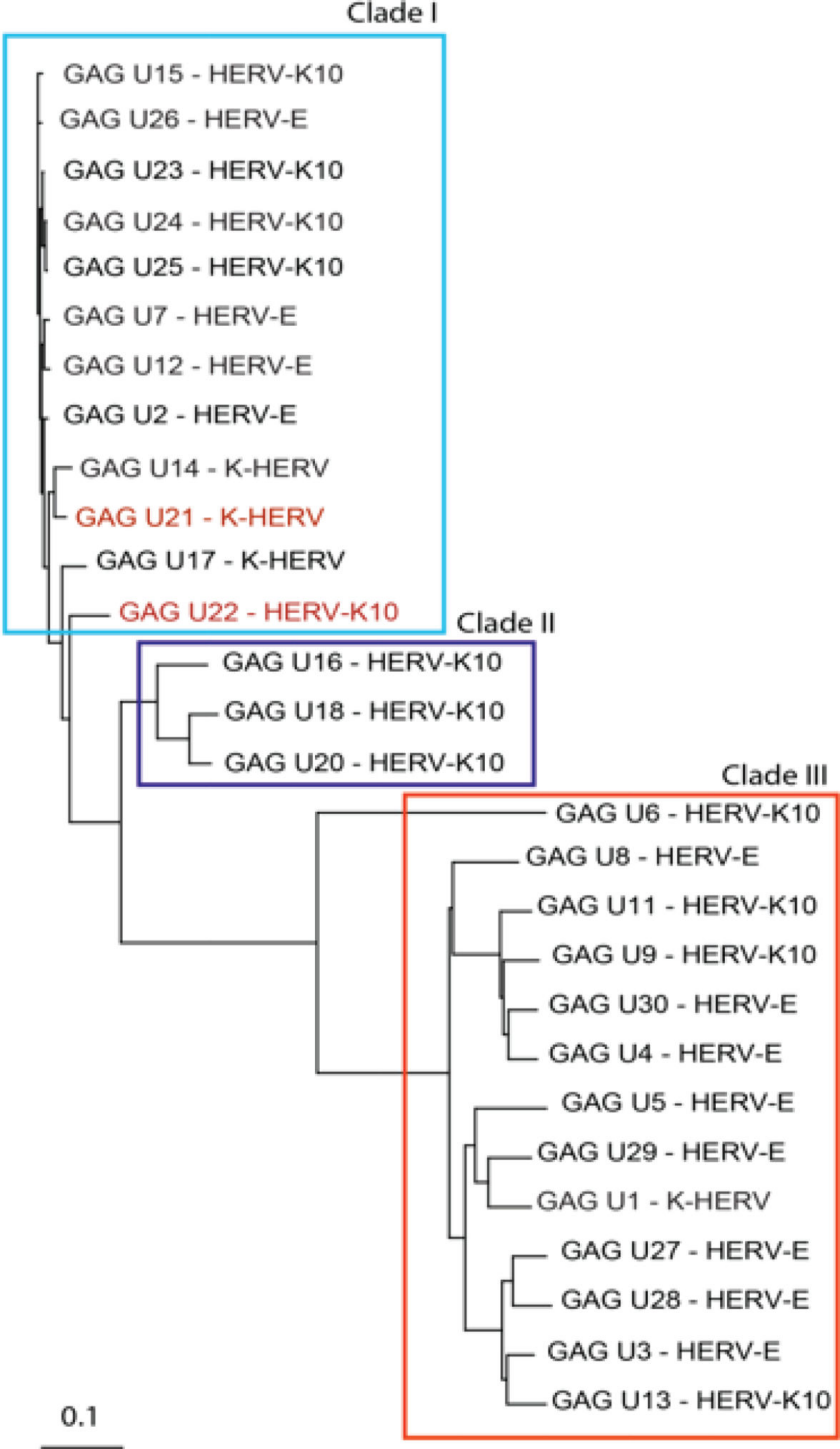
GAG sequence phylogeny-Twenty-eight non redundant GAG sequences were pulled from the RVGC (see Methods). Phylogeny was constructed using a neighbor-joining algorithm from the distance matrix. GAG sequences were classified into three different clades outlined by the boxes. GAG sequences over expressed in MS compared to controls and as determined by RNA-seq are displayed in red font.

**Figure 2:**
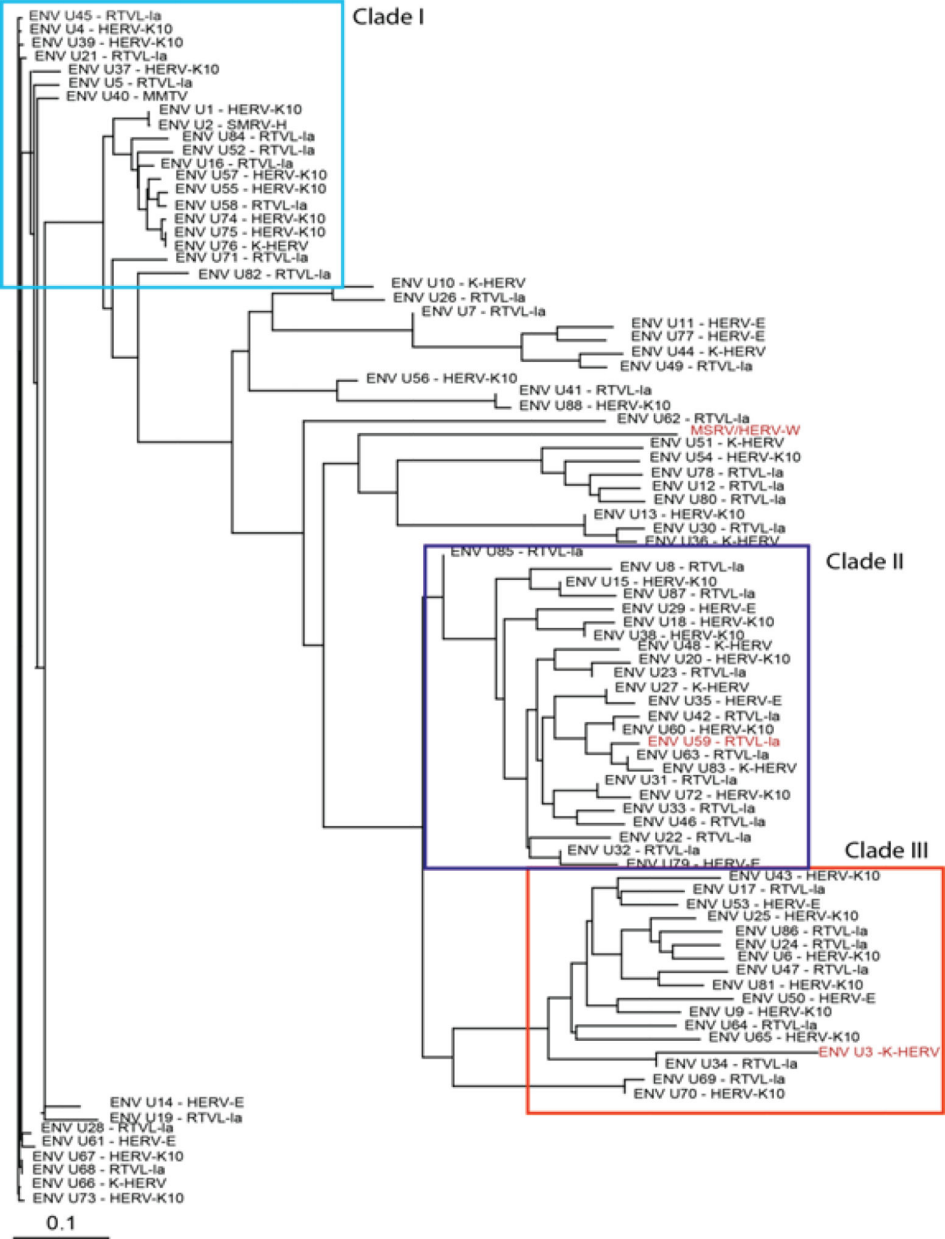
ENV sequence phylogeny-Eighty-eight non redundant ENV sequences were pulled from the RVGC (see Methods). Phylogeny was constructed using a neighbor-joining algorithm from the distance matrix. ENV sequences were classified into three different clades outlined by the boxes. ENV sequences over expressed in MS compared to controls and as determined by RNA-seq are displayed in red font.

Deep sequencing showed that some HERV domains including GAG and ENV were overexpressed in MS samples [20,21]. Based on these results, the 28 GAG and 88 ENV gene sequences in the RVGC were aligned using Clustal Omega [23]. The direction of each sequence was determined by finding the longest open reading frame of the sequence. Before alignment all domain sequences were rectified to the sense orientation if necessary. GAG and ENV phylogenies were constructed using neighbor-joining (Figures 1 and 2). The major nodes within the phylogenies were used to define clades within the GAG and ENV domains (Figures 1 and 2).

In order to amplify multiple similar GAG and ENV loci spread across the human genome, primers were designed to be clade-specific, rather than locus specific. For this purpose, clade consensus sequences from the Clustal Omega multiple alignments were used to derive qPCR primer pairs, using Primer3 [24]. These primer pairs were 20–24 bp in length, with annealing temperatures of 58–60°C, with no predicted low-energy self-dimers, predicted to give 100–120bp amplicons. The primer sequences were aligned to the human genome using BLAST to predict possible amplicons [25]. Primers predicted to specifically bind the clade-specific consensus sequence were selected to minimize their hybridization to the other clades. Several primer pairs were designed for each of the GAG and ENV clades. The primer sequences used in the study are specified in Table 2.

### c. RNA extraction and cDNA synthesis

Study specimens from the frozen blocks were obtained using 6 mm sterile circular skin biopsy punch blades (VWR, Radnor, PA, USA). The tissue specimens (each approximately 100 mg) were homogenized by finely chopping them with a sterile blade in a sterile weigh boat, all on dry ice, before placing them into RNA-lysis buffer. The RNA was extracted using the RN easy Lipid Tissue Mini Kit (Qiagen, **Hilden, Germany** and Germantown, MD, USA) and quantified using an RNA High Sensitivity (HS) Assay Kit (Life Technologies/Thermo Fisher Scientific, Carlsbad, CA, USA) on a Qubit 2.0 Fluorometer instrument (Invitrogen/Thermo Fisher Scientific, Carlsbad, CA, USA). Extracted RNA was DNase treated using a TURBO DNA-free Kit (Life Technologies/Thermo Fisher Scientific, Carlsbad, CA, USA). Nucleic acids in the DNase treated specimens were quantified by using a Qubitds DNA HS Assay Kit. A total of 500 ng of RNA was used for the reverse transcription (RT) reaction using Omniscript RT (Qiagen, Hilden, Germany and Germantown, MD, USA), using random hexamers according to the manufacturer’s instructions. The resulting cDNA was aliquoted and stored at −20°C.

Several reference genes, previously used in studies of gene expression in neuronal tissue, were considered for use in this study: ubiquitin C (UBC), RNA polymerase II polypeptide (RP II), ribosomal large subunit protein 19 (RPL19), β-2-microglobulin (B2M), and glyceraldehyde-3-phosphate dehydrogenase (GAPDH) [26,27]. RNA expression levels for each of the candidate reference genes were determined by qPCR of the MS and control specimens. The geNorm application was used to determine the best reference gene [28]. RPL19 was selected as the reference gene due to the least variation in expression levels among all the specimens.

Five primer pairs were designed from consensus sequences for each of the 3 GAG and 3 ENV clades, for a total of 30 primer pairs. Melting curves from qPCR reaction were examined to determine suitable primer pairs to carry forward in the study. Specifically, 12 primer pairs demonstrating single homogenous amplification products were used for the study. Standard curves for each primer pair including reference gene candidates are shown in supporting data.

### d. qPCR and Data analysis

Each reaction was run in duplicate with no-template, and no-RT controls. Average Ct value was considered for analysis according to the MIQE guidelines [29]. Control and MS samples (5 µl each) were pooled together and serially diluted 2-fold. This dilution series was used to obtain Ct standard curves for each gene of interest (Supplementary Data). Calibration curves with slopes in the range of .95–1.29 and R^2^<0.95 were considered for analysis. For each plate, PCR amplification efficiency was determined from the standard curve using the equation: PCR efficiency=(2^−1/slope^−1) × 100% (Supplementary Figures). Gene expression values were calculated from the standard curve for each control and MS specimen, normalized by RPL19 expression for each sample. The median expression values for each primer pair was derived for the control and MS groups. The expression change (fold change) was derived by dividing the median expression value of the MS group from the median of the control group. The Mann-Whitney U-test was used to detect differences among the groups [30]. We considered the difference to be statistically significant when p< 0.05.

## Results

### a. Frozen Brain samples

Characteristics of the study samples are shown in Table 1. Thirty-three MS samples were compared to 9 normal controls. The number of control specimens was lower than MS samples due to limited availability of normal controls from the brain bank sources. The post-mortem interval (PMI), i.e., the time between death and collection of the brain sample, range was 2–39 hours. The PMI was significantly longer in the MS group (μ=17 hrs) than in the controls (μ=9.5 hrs, p=0.0002). Age, sex, and year of collection were not significantly different between the groups.

### b. HERV GAG and ENV Phylogenies

Figure 1 shows the related GAG sequences classified by phylogeny into three different clades. According to Gypsy database annotation these clades include GAGs which belong to family or subfamily HERV-K10, K-HERV and HERV-E (specified in Table S1). Phylogeny using a neighbor joining tree of the ENV sequences also revealed three separate clades (Figure 2). Env sequence of HERV-W family; MSRV/HERV-W (AF331500.1), was also included in the ENV phylogeny [31]. The ENV sequences belonged to families including HERV-K10, K-HERV, HERV-E, Retrovirus-like Element-Isoleucine (RTVL-Ia), Mouse Mammary Tumor Virus (MMTV), and the D-type retrovirus Squirrel Monkey-like Retrovirus (SMRV-H) characterized from human B lymphocytes [32,33] (Table S1).

### c. Expression analysis

Twelve clade-specific primer pairs were tested for the GAG and ENV domains. The MSRV-ENV primer sequences were kindly provided by H. Perron [32]. Each primer pair was tested by performing qPCR with on the pooled RNA extractions (from all control and MS samples). *In silico* analysis of these primer pairs was performed using Mega BLAST. This showed a heterogeneous population of predicted amplicons that mapped to multiple different human chromosomal locations specified in Table S1.

**Figure 3:**
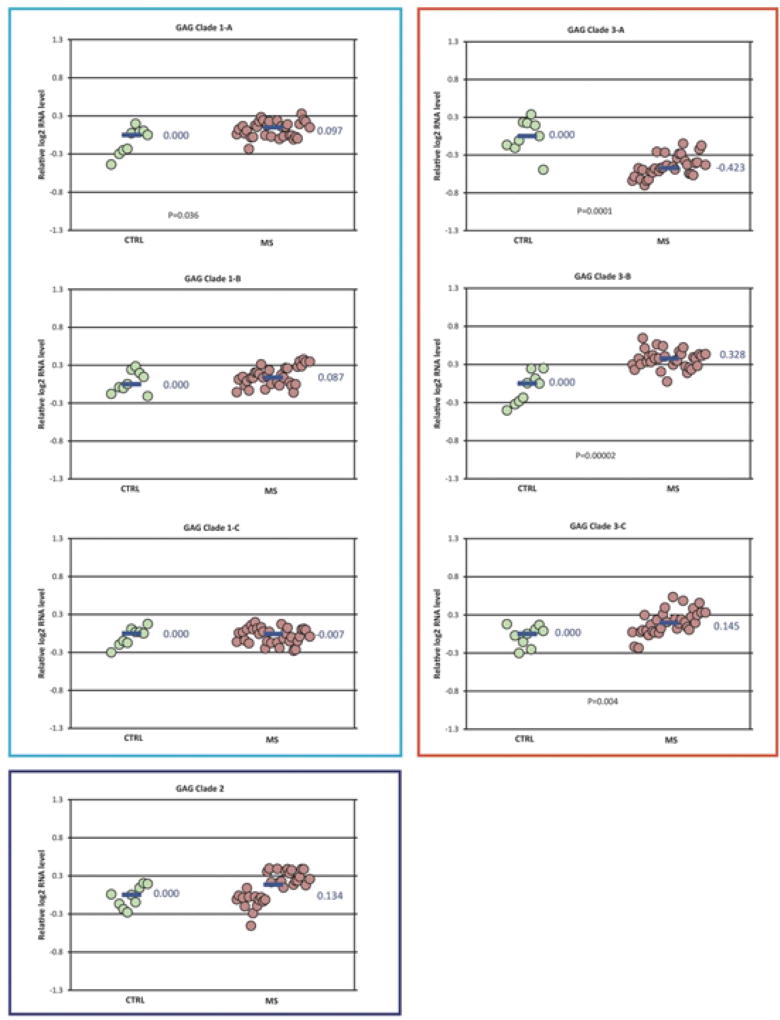
GAG domain expression in MS *vs* control-Differences in normalized expression for each primer pair are shown as Log_2_ (MS median/control median). Significance in expression differences was determined using the nonparametric Mann-Whitney test comparing expression values for the MS patients (N=33) to the controls (N=9). p-values for the statistically significant comparisons are shown on the chart.

RPL19 was selected as the reference gene for normalization of expression because it had the lowest expression variation among the samples. Normalized gene expression levels for each of the targeted HERV GAG domains are shown in Figure 3. The data is summarized as relative fold change (log_2_) for each primer pair. Expression of GAG clade 1A, 3B, and 3C were significantly increased while GAG clade 3A was decreased in the MS samples (N=33) compared to controls. The pattern of GAG expression for the MS subtypes among each of the clades was similar to that for the MS group as a whole (Figure S1). The magnitude of the significant changes in GAG expression was small with fold-changes only 0.74–1.25.

**Figure 4:**
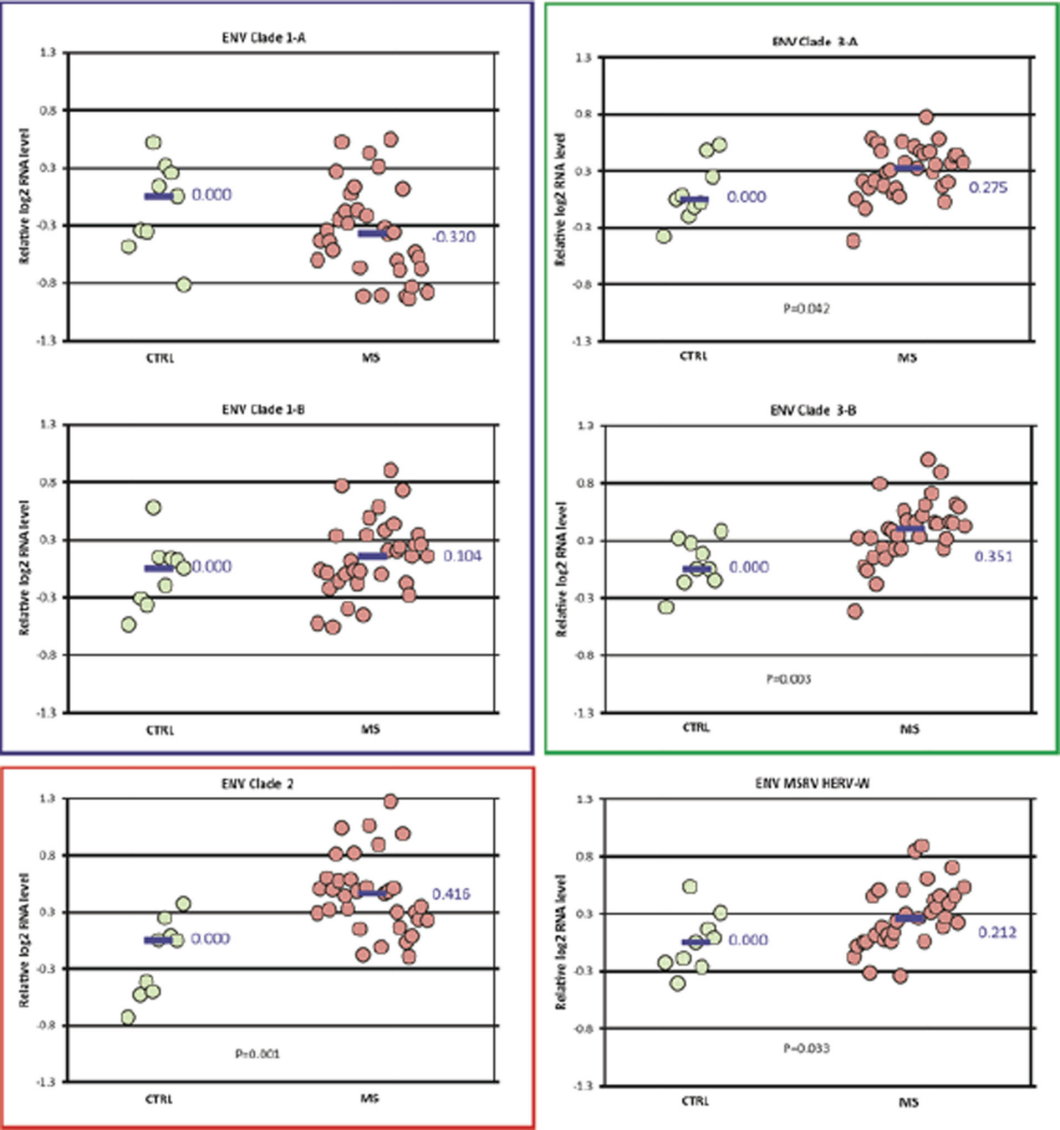
ENV domain expression in MS *vs* control-Differences in normalized expression for each primer pair are shown as Log_2_ (MS median/control median). Significance in expression differences was determined using the nonparametric Mann-Whitney test comparing expression values for the MS patients (N=33) to the controls (N=9). p value for statistically significant groups is mentioned.

Expression differences between the MS and control groups among the ENV clades studied were also small, with fold-changes less than 1.5 (Figure 4). No differences were seen in the expression of ENV clade 1. The expression of ENV clade 2 (fold-change 1.33), clades 3A and 3B (fold-change 1.2–1.27), and ENV MSRV/HERV-W (fold-change 1.15) were all increased in the MS group compared to controls. The pattern of ENV expression for the MS subtypes among each of the clades was similar to that for the MS group as a whole (Figure S2).

## Discussion

This study compared HERV GAG and ENV domain expression measured by qPCR in frozen brain tissue specimens collected from individuals with MS compared to neurologically normal controls. While this study showed several significant differences in HERV expression, the magnitude of these differences was relatively small (less than 1.5-fold). These results indicate that the expression of HERV GAG and ENV regions do not differ greatly between different types of MS and controls in these frozen brain samples.

There are approximately 40,000 HERV elements distributed among thirty five human HERV families classified by Repbase [14,34]. Transcriptional activity of representative members of 20 HERV families in several different normal human tissues has been studied by microarray [34]. Our attempts to clone and amplify specific HERV loci of interest from the cDNA derived from these frozen brain samples was not successful, likely due to the highly homologous nature of the HERV sequences we were trying to study (data not shown). For this reason, a more unconventional approach was employed utilizing alignments among related HERVs (clades).

Specifically, this study used an alignment approach, employing HERV phylogenies (Figures 1 and 2) to design consensus primer sequences (Table 2) amplifying multiple different HERV ENV and GAG regions. These regions, designated as clades and subclades in this study, were derived from 28 GAG and 88 ENV domain sequences distributed among many different HERV families (Table S1). The approach we took does not formally exclude the possibility that some very specific HERV loci might very elevated in MS brain tissue. Rather, we were interested in getting a broad overview of HERV expression in MS brain tissue, complementing our previous sequencing data.

HERVs constitute about 8% of the human genome [35]. They are scattered widely throughout the human genome, and most cannot produce virus particles. HERVs have recently been shown to act as tissue-specific enhancers of gene transcription [36], and some human proteins are the result of HERV “domestication.” One example is syncytin, an important protein that allows for development of the placenta [37,38]. HERVs have been proposed as etiological cofactors in chronic diseases such as cancer, autoimmune disorders, and neurological disease [39]. Two complete ENV ORFs from the HERV-W family are thought to be associated with MS: MSRV, and ERVWE1 (syncytin-1) [10].

Our group recently reported an RNA-seq study showing that some HERVs and other retro elements are significantly over expressed in a smaller set of frozen MS and control brain tissue samples. That study found over expression of GAG sequences mapping to GAG Clade 1 (HERV-K10, K-HERV) and ENV sequences mapping to ENV clades 2 (RTVL-Ia) and 3 (K-HERV) (Figures 1 and 2). However, these findings were partially confirmed by the present qPCR study where expression of ENV clades 3A and 3B (HERV-K and HERV-E) were increased in MS. Other groups have reported increased levels of ENV MSRV (HERV-W) in blood, spinal fluid, and brain samples in MS patients [17]. We did confirm that finding here, but the magnitude of the MSRV expression increase was relatively small – only 1.15 fold-change).

A number of assumptions are inherent to this study. First, HERV GAG and ENV regions were chosen for evaluation because multiple reports show that these are the retrovirus genes most likely to be involved in immunopathogenesis [17,40–43]. Second, we assume that the collapse of GAG and ENV genes from the Gypsy 2.0 database into the RVGC is sufficiently representative of all GAG and ENV HERV domains. Third, we assume that the primer pairs designed from the HERV GAG and ENV phylogenies and divided into related sequences (clades), providing a reasonable representation of these domains’ RNA within the samples. Finally, we assume that these postmortem samples, typically taken several hours after death, accurately reflect the premortem RNA content of the tissue. There is no doubt that cellular RNA is not static after death, and this clearly a limitation of our study, but fresh brain samples from neurologically-normal or diseased people are simply not available for comparison. Our MS samples had significantly longer PMIs than the controls, and this could have decreased the expression measured in the MS group more than in the controls, leading to an underestimation of expression differences between the groups. However, any PMI effect on expression should be canceled by RPL19 normalization which, presumably, deteriorates at a similar rate to the HERV domains we measured. All these assumptions can be criticized, but the goal of the study was to provide a broad view of HERV expression in MS white matter samples.

## Conclusion

Expression of GAG and ENV mapping to HERV-E, RTVL and HERV-K10 families were significantly increased in the MS group. However, the relative expression differences between the MS and control groups were small, differing less than 1.5-fold. These results indicate that the expression of HERV GAG and ENV regions do not differ greatly between MS and controls in these frozen brain samples. This work only partially confirms the sequencing data previous derived from similar frozen MS brain specimens.

## Supplementary Material

Details qPCR

Figures S1 and S2

Predicted Amplicons

Table S1

qPCR std

## Figures and Tables

**Table 1:** Characteristics of the frozen brain samples used in this study.

Group	Specimen	PMI	Age	Sex	Collection Year	Clinical History/MS Type	Specimen Location
Normal Controls	5	2	55	F	1989	NA	cerebrum
	202*	4	57	M	1983	post-surgical infection	cerebrum
	214*	4	56	M	1981	heart disease	cerebrum
	3236	16	67	M	2002	esophageal cancer	frontal WM
	3276*	19	54	M	2002	heart disease	frontal WM
	33*	5	69	M	1983	lung disease	cerebrum
	3371*	16	52	M	2002	lung cancer	frontal WM
	3348*	9	76	F	2002	heart disease, diabetes	frontal WM
	3540	11	68	M	2003	lung cancer	frontal WM
MS	159	NA	53	M	1983	chronic progressive	WM
	164	NA	NA	M	1983	Unclassified	WM
	216	5	71	F	2003	Unclassified	WM
	2096	NA	NA	M	NA	secondary progressive	periventricular WM
	2485*	9	69	M	1997	primary progressive	frontal WM
	2696*	21	86	F	1998	primary progressive	periventricular WM
	2742	17	60	M	1998	primary progressive	periventricular WM, cerebellum
	2743	23	59	F	1998	secondary progressive	subcortical WM
	2758	NA	60	M	1999	secondary progressive	periventricular WM
	2778	39	61	F	1998	secondary progressive	WM, temporal cortex
	2800	9	64	F	1998	secondary progressive	WM, frontal cortex
	2946*	NA	59	M	1999	secondary progressive	periventricular WM, parietal lobe
	2966	NA	55	F	2000	secondary progressive	periventricular WM
	3010	NA	49	F	2000	secondary progressive	frontal WM, frontal lobe
	3056	10	61	M	2000	relapsing remitting	WM, frontal cortex
	3093	18	68	M	2000	Unclassified	periventricular WM, basal ganglia
	3161*	20	51	F	2001	secondary progressive	WM, frontal-parietal
	3250	17	75	M	2002	secondary progressive	periventricular WM
	3289	29	54	F	2001	Unclassified	periventricular WM
	3413	10	38	F	2002	relapsing remitting	periventricular WM
	3422	12	62	M	2002	relapsing remitting	periventricular WM, cerebellum
	3474	9	70	F	2002	chronic progressive	periventricular WM
	3502	16	78	M	2003	secondary progressive	periventricular WM,
	3509*	11	74	F	2003	primary progressive	periventricular WM
	3831	21	82	F	2004	chronic progressive	occipital WM
	3835	29	65	M	2004	chronic progressive	periventricular WM
	3867	13	75	M	2004	secondary progressive	periventricular WM
	3891	25	53	M	2005	chronic progressive	occipital WM
	3894	32	71	M	2004	secondary progressive	parietal WM
	3928	10	53	F	2004	secondary progressive	occipital WM
	4201	14	75	F	2006	Unclassified	occipital WM
	4212	19	50	F	2006	Unclassified	frontal WM
	4218	15	63	F	2006	Unclassified	frontal WM

* Specimen used in the preceding RNA sequencing study [21].

NA=information not available from the participating brain banks PMI=post-mortem interval (i.e., time elapsed between death and collection of the tissue specimen), rounded to the nearest hour

**Table 2:** Primers designed for this study.

Domain	Primer Pair	Forward sequence (5’-3’)	Reverse Sequence (5’-3’)
GAG	Clade 1A	GCAACATCTTGGAGCCTTG	ATCTGCCTTAGAGCCTGGGA
	Clade 1B	ATCTTGGAGCCTTGCCACAA	ATCTGCCTTAGAGCCTGGGA
	Clade 1C	AACATCTTGGAGCCTTGCCA	ATCTGCCTTAGAGCCTGGGA
	Clade 2	GCCTTCACATATTCTGTAATT	GCAAGGTTGCAAGATGCAGCTC
	Clade 3A	CAAATGCCCCTGAGAGAGCA	TTCCAGTTGAGAAGGTCGGC
	Clade 3B	CCACCAGATAACCACACCCC	TGCTCTCTCAGGGGCATTTG
	Clade 3C	TTAAGAGGACAGGCAGCAGC	GGGGTGTGGTTATCTGGTGG
ENV	Clade 1A	TGGGAAAACAGAATGGCCCT	AAGGCCCTTGTTATGCTCCC
	Clade 1B	TCCATGGACTGACAGTGGGA	AAAGCAATTCCAGCAGCCAC
	Clade 2	AGACCCACCAGTCAAATGGC	TTTGGTGTGATCCCACCTGG
	Clade 3A	GCGGTAAGTCTTTGCAAGGC	CCAGAATGGCTCCAGACATGT
	Clade 3B	CTGGACAATCAGGACCCACC	GGCCTTGCAAAGCCTTTGTT
	MSRV	CTTCCAGAATTGAAGCTGTAAAGC	GGGTTGTGCAGTTGAGATTTCC
Control	RPL19	ATGTATCACAGCCTGTACCTG	TTCTTGGTCTCTTCCTCCTTG
	RPL13	CCTGGAGGAGAAGAGGAAAGAGA	TTGAGGACCTCTGTGTATTTGTCAA
	UBC	ATTTGGGTCGCGGTTCTTG	TGCCTTGACATTCTCGATGGT
