# Supplementary material for: Analysis of Human Endogenous Retrovirus Expression in Multiple Sclerosis Plaques
Source: J Emerg Dis Virol. Author manuscript; Available in PMC 2017 Sep 1. (PMC5580941; doi:10.16966/2473-1846.133)
Supplement: Details qPCR [file NIHMS900509-supplement-Details_qPCR.docx]

**Supplementary Data**

Details of qPCR experiments

qPCR experiments conform to the MIQE guidelines as recommended by:

Bustin SA, Benes V, Garson JA, Hellemans J, Huggett J, et al. (2009) *The MIQE guidelines: minimum information for publication of quantitative real-time PCR experiments.* Clin Chem 55: 611-622.

EXPERIMENTAL DESIGN

- Definition of experimental and control groups:

Cryopreserved normal white matter control brain specimens from patients who did not had any brain diseases (N=9), and Cryopreserved white matter from MS plaques (N=33) and were obtained from the Rocky Mountain MS Center Tissue Bank (Westminster, CO) and the UCLA Human Brain and Spinal Fluid Resource Center (Los Angeles, CA) (Please see Table 1). Number within each group:

- Control group = 9 and Experimental group = 33 [chronic progressive (n=5), primary progressive (n=4), secondary progressive (n=14), relapsing remitting (n=3) and unclassified confirmed MS cases (n=7)].
- Assay carried out by core lab or investigator's lab? : investigator’s lab

SAMPLE

- Description: Volume/mass of sample processed: approximately 100 mg specimens of frozen brain were used for RNA extractions
- Processing procedure: The samples were received frozen from the brain banks. A cold chain was maintained such that the samples were never thawed until being prepared for homogenization. This was performed by chopping the tissue on dry ice and making fine paste. Then the homogenized tissue was added to a Qiagen lysis buffer with four 20 second pulses on a vortex.

NUCLEIC ACID EXTRACTION

- Procedure and/or instrumentation : Name of kit and details of any modifications: RNeasy Lipid Tissue Mini Kit (Qiagen, **Hilden, Germany** and Germantown, MD)
- Source of additional reagents used: QiA lysis buffer
- Details of DNase or RNAse treatment: TURBO DNA-free™ Kit (Life Technologies, CA).
- Nucleic acid quantification & Contamination assessment (DNA or RNA): RNA was quantified by Qubit® RNA HS Assay Kit and on Qubit® 2.0 Fluorometer instrument (Invitrogen/Thermo Fischer [Scientific](https://www.google.com/url?sa=t&rct=j&q=&esrc=s&source=web&cd=2&cad=rja&uact=8&ved=0ahUKEwjq2qaKmsPLAhVPxmMKHdKgBV8QFggsMAE&url=https%3A%2F%2Fwww.thermofisher.com%2F&usg=AFQjCNFb0eNGbOXEXAPGjboiMZvISmbgTw&sig2=DOVT9M9LfHPGrGALyTu76Q&bvm=bv.116954456,d.cGc), [Carlsbad, CA](https://www.google.com/search?safe=active&espv=2&biw=1680&bih=881&q=Carlsbad+California&stick=H4sIAAAAAAAAAOPgE-LSz9U3MKmqSInPVeIAsYtMyvO0tLKTrfTzi9IT8zKrEksy8_NQOFYZqYkphaWJRSWpRcUA_pIQXEQAAAA&sa=X&ved=0ahUKEwjq2qaKmsPLAhVPxmMKHdKgBV8QmxMIggEoATAO), USA). DNA in the prep was quantified by Qubit® dsDNA HS Assay Kit.
- Yield : 5-30 µg RNA was extracted from the samples.
- Inhibition testing (Ct dilutions, spike or other): The samples were pooled and used in series two fold of dilution to plot standard graph.

REVERSE TRANSCRIPTION:

- Per instruction manual of Omniscript RT kit (Qiagen) Cat No./ID 205111
- Complete reaction conditions for RT: Amount of RNA and reaction volume: A total of 500ng of RNA from each sample was used for RT reaction. The reverse transcription (RT) reaction was performed using Omniscript RT (Qiagen, Hilden, Germany and Germantown, MD)
- Priming oligonucleotide concentration: random hexamers were used in a final concentration of 10 μM in the reverse-transcription reaction.
- Reverse transcriptase components and concentrations in reaction:

| **Component Final concentration of** | **Volume/reaction** | **Final concentration of Master mix** |
| --- | --- | --- |
| 10x Buffer RT | 2 μl | 1x |
| dNTP Mix (5 mM each dNTP) | 2 μl | 0.5 mM each dNTP |
| Random Hexamer primer (10 μM) | 2 μl | 1 μM |
| RNase inhibitor (10 units/μl) | 1 μl | 10 units (per 20 μl reaction) |
| Omniscript Reverse Transcriptase | 1 μl | 4 units (per 20 μl reaction) |
| RNase-free water | 7 μl | variable |
| Template RNA | 5μl | 500ng |
| Total Volume | 20 μl |  |

- Temperature and time: 59 for 30 s
- Storage conditions of cDNA : -20˚C

qPCR TARGET INFORMATION:

- Sequence accession numbers: provided in supplementary table 1
- Location of amplicons: provided in supplementary Table S2
- Amplicon length : 120-130 bp
- In silico specificity screen (BLAST, etc) : Yes, Blast against human genome build 37.p13
- Pseudogenes, retropseudogenes or other homologs? : Due to the redundancy and duplication of retroviral sequences (GAG and ENV) in NCBI, a HERV phylogeny approach was used to derive primers that bind to many similar loci produced throughout the genome. An in silico analysis of the expected amplicons using these primers was performed before proceeding with in vitro experiments and given in Table S2.
- Secondary structure analysis of amplicon: Primers were designed using Primer3 (primer3.sourceforge.net/). Amplicons with “No Secondary Structures” were selected.
- qPCR OLIGONUCLEOTIDES : 20-24 bp in length (sequences are specified in Table 2)

qPCR PROTOCOL :

- Complete reaction conditions: Reaction volume and amount of cDNA/DNA: cDNA of MS and control samples was diluted to 1: 100 and 5ul from each sample was used as a template in total reaction volumes of 20 µl containing Fast SYBR Green mix
- qPCR kit used : Fast SYBR Green mix (Roche Diagnostics, Indianapolis, IN, USA) Cat number 04707516001.
- Buffer and dNTP concentration : Per manufacturer’s instructions
- Primer concentrations: 0.3 µM of forward and reverse primers
- Real time PCR conditions: Real-time qPCR was performed using 45 cycles (95°C for 30 seconds, 58-60°C for 30 seconds, 72°C for 1 minute) on a Roche LC480 instrument, and fluorescence was measured after each of the repetitive cycles. Conditions were optimized to amplify different GAG loci in one reaction. Experiments were performed with different primer pairs for each GAG and ENV clade.

qPCR VALIDATION :

- Evidence of optimization (from gradients) : Control and MS samples (5µl each) were pooled together and serially diluted with two fold dilutions. This dilution was used to obtain Ct standard curves for each gene of interest. Standard Curve graphs for each gene of interest are shown below:
- Example of showing Ct values of the Pool Dilution (1:05, 1:10, 1:20, 1:40 , 1:80, 1:160, 1:320 and 1:640)

Pool Dilution: Control and MS samples (5µl each) were pooled together and serially diluted with two fold dilutions.


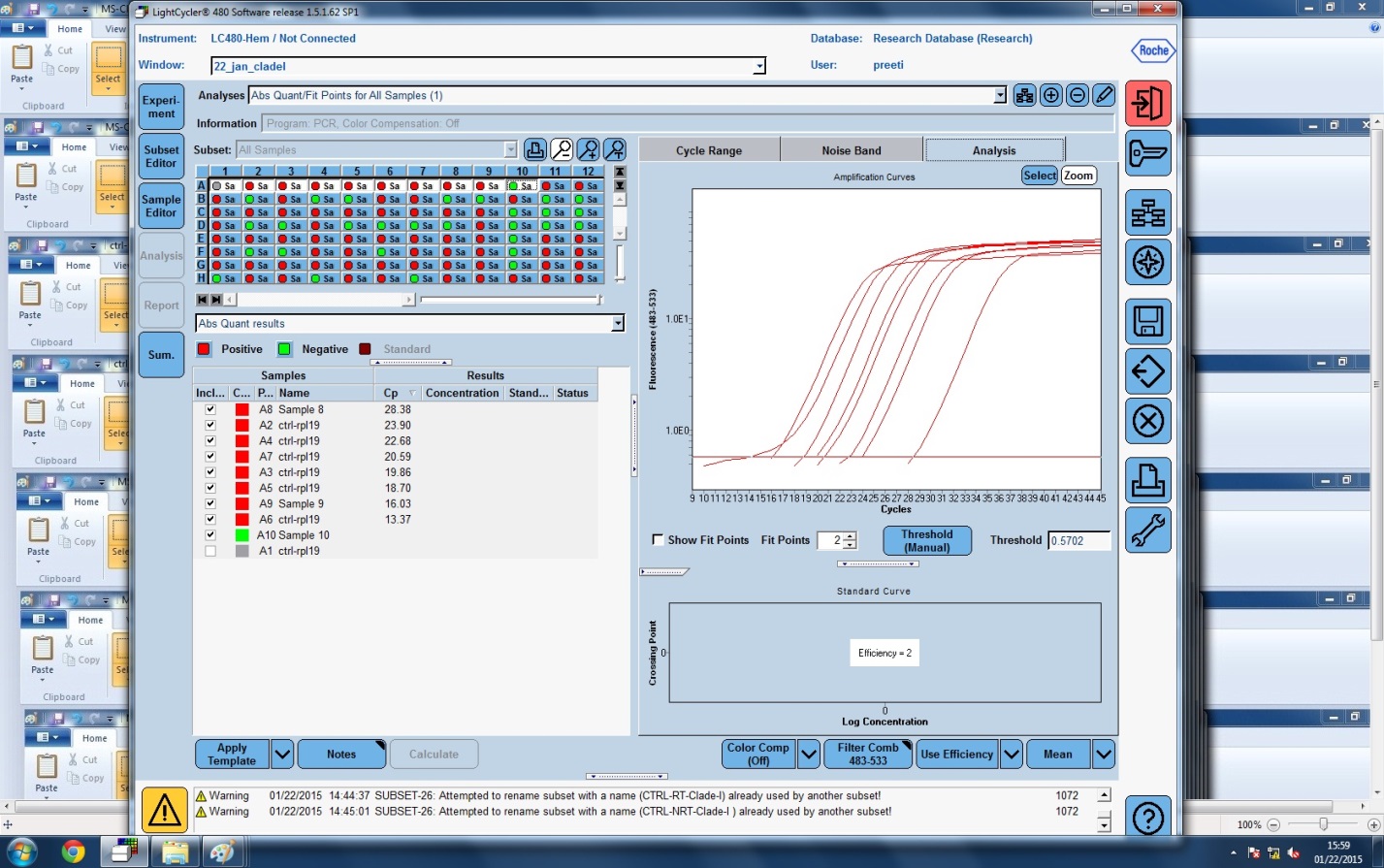


- Above, is a snap shot of a representative standard curve based on pool dilutions for one primer pair (Reference genes and GAG clade primers). Standard curves for each primer pair are given in the supplementary figures.
- Specificity: Dilutions resulting into Ct values <40 were considered as cut-off. Calibration curves with a slope in range of .95-1.27 (according to 2 fold dilution) and R^2^ <0.95 were considered for analysis. Based on the dilution, gene expression values were calculated from the standard curve for each control and MS specimen.
- Ct of the no template control (NTC) : > 42
- Standard curves with slope and y-intercept: Using the linear equation y= mx +c; where m is slope and c is Y intercept which is concentration of cDNA dilution converted into log2 scale. Y is the Ct Value and x is the arbitrary concentration value. Gene expression values of unknown (MS and Control) was derived by using the Ct Value from each of these reactions.
- Table showing the Dilution and arbitrary concentration value of dilution in log2.

| Dilution | 1:05 | 1:10 | 1:20 | 1:40 | 1:80 | 1:160 | 1:320 | 1:640 |
| --- | --- | --- | --- | --- | --- | --- | --- | --- |
| Unit value | 0.2 | 0.1 | 0.025 | 0.0125 | 0.00625 | 0.00312 | 0.00156 | 0.00078 |
| Log2 Dilution | -2.32193 | -3.32193 | -5.32193 | -6.32193 | -7.32193 | -8.32193 | -9.32193 | -10.3219 |
| Expression value x=(y-c)/m | 2.23049 | 3.23485 | 4.23920 | 5.24355 | 6.24790 | 7.25226 | 8.25661 | 9.26096 |

- Slope Value m, was 0.95 - 1.29 for the 2-fold dilutions on each plate (see Supplementary Figures, slide 11).
- PCR efficiency calculated from slope: For each plate PCR amplification efficiency was determined from standard curve using the slope using equation: PCR efficiency= (-1 + 2^-1/slope^) x 100%. (detailed in Supplementary Figures, slide 11)
- Confidence interval for PCR efficiency or standard error
- r2 of standard curve : .95-.99.
- Evidence for limit of detection: Standard graph from each primer pair with Pooled sample dilution. Lowest Ct Value for 1:05 dilution was 17.

DATA ANALYSIS

- qPCR analysis program (source, version) : LightCycler® 480 Software, Version 1.5**.** Ct values were plotted in Excel. Expression values were calculated using the standard curve formulas for each gene of interest.
- Ct method determination: Per LightCycler® 480 Software, Version 1.5
- Outlier identification and disposition : Per As per LightCycler® 480 Software, Version 1.5
- Results of NTCs: > 42 Ct value
- Justification of number and choice of reference genes: ubiquitin C (UBC), RNA polymerase II polypeptide (RPL-19), β-2-microglobulin (B2M), and glyceraldehyde-3-phosphate dehydrogenase (GAPDH) were used as reference genes. Gene expression among the total MS sample and also each subtypes and control specimens were determined by qPCR for each of the candidate reference genes. The geNorm application was used to rank the least variable candidate reference gene. We identified RPL19 as the best reference gene for the data-set, as it had the lowest expression variation among all the samples we evaluated.
- Description of normalization method: The relative target gene expression was normalized with the reference gene RPL-19. The median expression value for each gene/primer set was derived for the control and MS groups. The expression changes in terms of Fold change (log2) for each primer set were derived by dividing the median expression value of the MS group by the median of the CTRL group.
- Number and concordance of biological replicates: Duplicates
- Number and stage (RT or qPCR) of technical replicates: Duplicates
- Statistical methods for result significance: Mann-Whitney tests were performed on the dataset to detect differences among the variables. We considered the difference to be statistically significant when p < 0.05.
- Software (source, version) : http://vassarstats.net/
