# Supplementary material for: Analysis of Human Endogenous Retrovirus Expression in Multiple Sclerosis Plaques
Source: J Emerg Dis Virol. Author manuscript; Available in PMC 2017 Sep 1. (PMC5580941; doi:10.16966/2473-1846.133)

**Fig S1. GAG domain expressions in Brain Tissues.** Differences in normalized expression for each primer pair are shown as Log_2_. Significance in expression differences was determined using the Mann-Whitney test comparing expression values for the Control patients (N=9) to the Chronic Progressive MS [CPMS (N=5)], primary progressive MS [PPMS, N=4], secondary progressive [SPMS, N=14], relapsing remitting MS [RRMS, N=3] and unclassified MS (N=7). Statistically significant groups (p < 0.05) are noted by *.


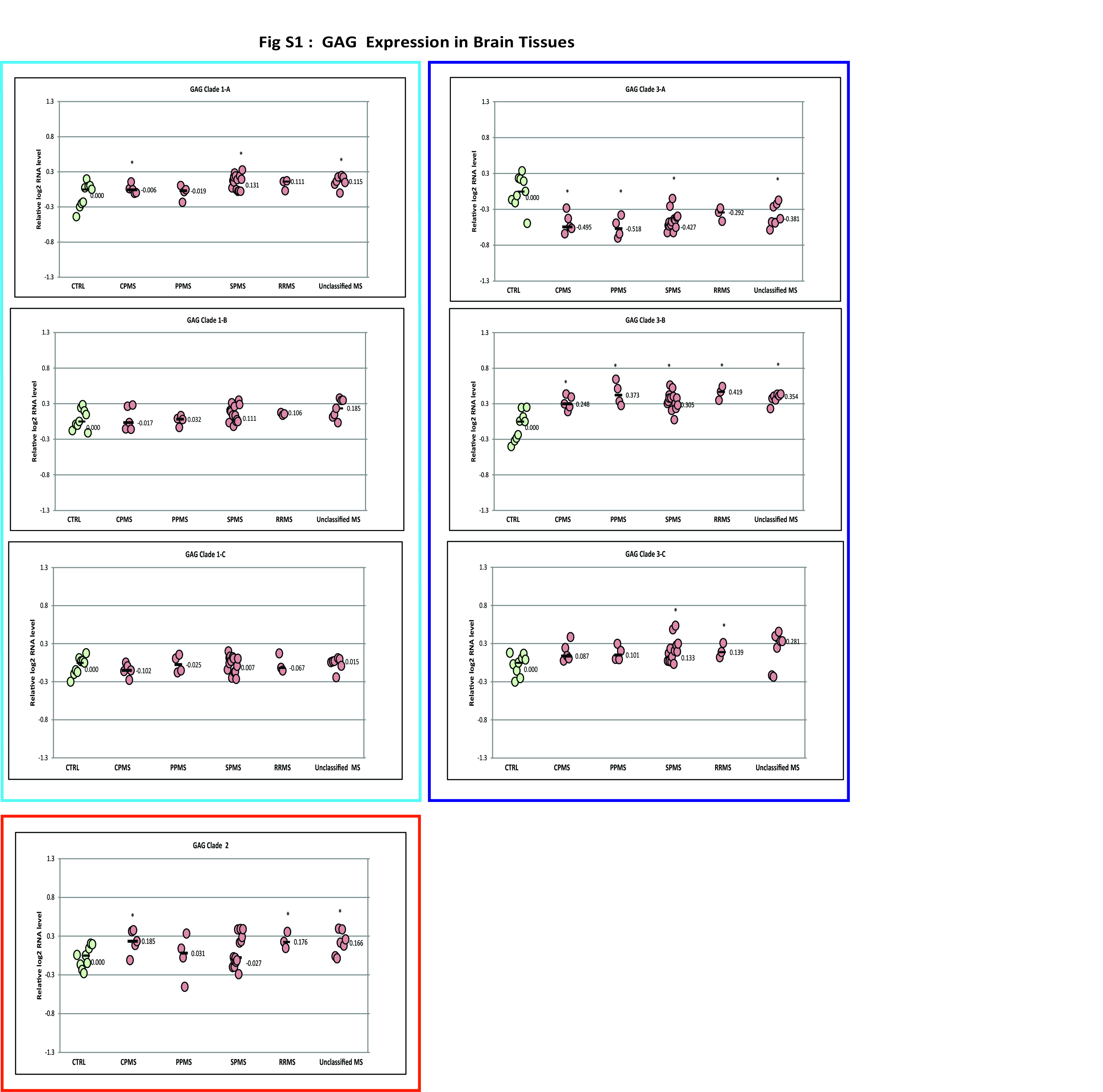


**Fig S2. ENV domain expressions in Brain Tissues.** Differences in normalized expression for each primer pair are shown as Log_2_. Significance in expression differences was determined using the Mann-Whitney test comparing expression values for the Control patients (N=9) to the Chronic Progressive MS [CPMS (N=5)], primary progressive MS [PPMS, N=4], secondary progressive [SPMS, N=14], relapsing remitting MS [RRMS, N=3] and unclassified MS (N=7). Statistically significant groups (p < 0.05) are noted by *.


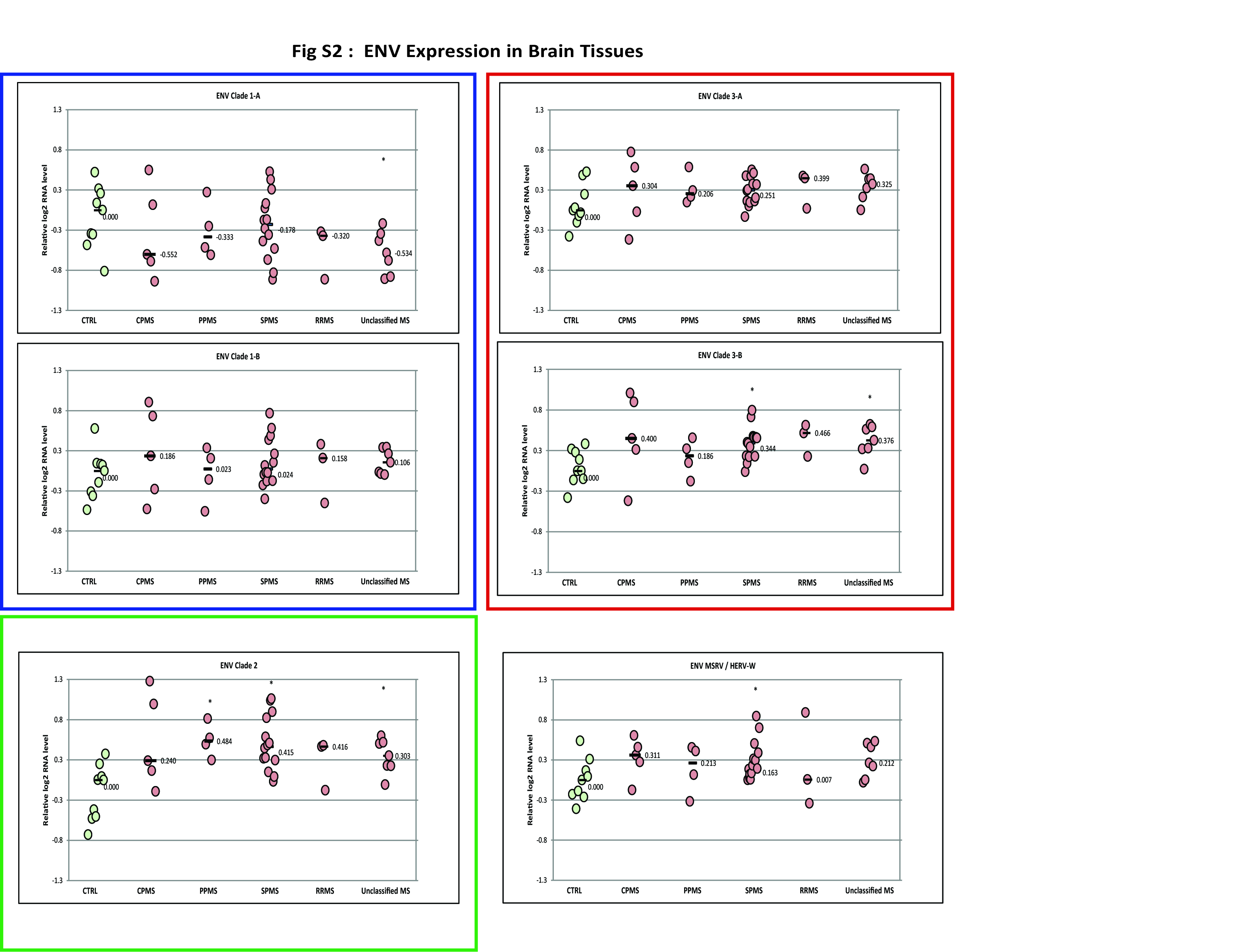

Supplement: Figures S1 and S2 [file NIHMS900509-supplement-Fig_S2_S2.docx]
