# Supplementary material for: Analysis of Human Endogenous Retrovirus Expression in Multiple Sclerosis Plaques
Source: J Emerg Dis Virol. Author manuscript; Available in PMC 2017 Sep 1. (PMC5580941; doi:10.16966/2473-1846.133)
Supplement: Table S1 [file NIHMS900509-supplement-Table_S1.docx]

**Table S1: HERV sequences used to develop qPCR primers.**

| Domain | U Number^1^ | Gypsy Recognition Domain^2^ | gi^3^ | Start Nucleotide^3^ | Length (bp) |
| --- | --- | --- | --- | --- | --- |
| GAG | 1 | K-HERV | 224589800 | 20481865 | 816 |
| GAG | 2 | HERV-E | 224589800 | 75844419 | 1287 |
| GAG | 3 | HERV-E | 224589801 | 35258727 | 1224 |
| GAG | 4 | HERV-E | 224589802 | 60862212 | 1245 |
| GAG | 5 | HERV-E | 224589803 | 17450946 | 804 |
| GAG | 6 | HERV-K10 | 224589803 | 32095804 | 756 |
| GAG | 7 | HERV-E | 224589803 | 58727764 | 1287 |
| GAG | 8 | HERV-E | 224589804 | 41453173 | 1296 |
| GAG | 9 | HERV-K10 | 224589804 | 67972360 | 804 |
| GAG | 11 | HERV-K10 | 224589810 | 20699104 | 765 |
| GAG | 12 | HERV-E | 224589810 | 28135395 | 1287 |
| GAG | 13 | HERV-K10 | 224589811 | 71273545 | 765 |
| GAG | 14 | K-HERV | 224589813 | 19939279 | 1059 |
| GAG | 15 | HERV-K10 | 224589814 | 18927835 | 1287 |
| GAG | 16 | HERV-K10 | 224589814 | 23881091 | 1824 |
| GAG | 17 | K-HERV | 224589815 | 9893352 | 1917 |
| GAG | 18 | HERV-K10 | 224589815 | 75601277 | 1050 |
| GAG | 20 | HERV-K10 | 224589816 | 9661433 | 1023 |
| GAG | 21 | K-HERV | 224589817 | 30493375 | 1200 |
| GAG | 22 | HERV-K10 | 224589817 | 46006565 | 1563 |
| GAG | 23 | HERV-K10 | 224589818 | 78433157 | 1287 |
| GAG | 24 | HERV-K10 | 224589819 | 4628594 | 1287 |
| GAG | 25 | HERV-K10 | 224589819 | 4637097 | 1287 |
| GAG | 26 | HERV-E | 224589820 | 7361933 | 1287 |
| GAG | 27 | HERV-E | 224589820 | 11770051 | 765 |
| GAG | 28 | HERV-E | 224589820 | 11792634 | 762 |
| GAG | 29 | HERV-E | 224589820 | 35810060 | 933 |
| GAG | 30 | HERV-E | 224589823 | 59307943 | 1284 |
| ENV | 1 | HERV-K10 | 224589800 | 13465685 | 1008 |
| ENV | 2 | SMRV-H | 224589800 | 13686521 | 1008 |
| ENV | 3 | K-HERV | 224589800 | 66883587 | 564 |
| ENV | 4 | HERV-K10 | 224589800 | 75846956 | 1263 |
| ENV | 5 | RTVL-Ia | 224589801 | 6867069 | 1065 |
| ENV | 6 | HERV-K10 | 224589801 | 85879093 | 249 |
| ENV | 7 | RTVL-Ia | 224589802 | 3469637 | 735 |
| ENV | 8 | RTVL-Ia | 224589802 | 4484932 | 294 |
| ENV | 9 | HERV-K10 | 224589802 | 23014944 | 270 |
| ENV | 10 | K-HERV | 224589802 | 51354150 | 720 |
| ENV | 11 | HERV-E | 224589802 | 58771057 | 1188 |
| ENV | 12 | RTVL-Ia | 224589802 | 59958681 | 1467 |
| ENV | 13 | HERV-K10 | 224589802 | 61990042 | 234 |
| ENV | 14 | HERV-E | 224589802 | 62137132 | 945 |
| ENV | 15 | HERV-K10 | 224589802 | 71458171 | 1014 |
| ENV | 16 | RTVL-Ia | 224589802 | 71475117 | 750 |
| ENV | 17 | RTVL-Ia | 224589803 | 10056039 | 270 |
| ENV | 18 | HERV-K10 | 224589803 | 32180481 | 234 |
| ENV | 19 | RTVL-Ia | 224589803 | 34773426 | 804 |
| ENV | 20 | HERV-K10 | 224589803 | 48995457 | 192 |
| ENV | 21 | RTVL-Ia | 224589803 | 58722167 | 1263 |
| ENV | 22 | RTVL-Ia | 224589803 | 78878127 | 210 |
| ENV | 23 | RTVL-Ia | 224589804 | 69463668 | 213 |
| ENV | 24 | RTVL-Ia | 224589805 | 53643304 | 174 |
| ENV | 25 | HERV-K10 | 224589806 | 34447718 | 261 |
| ENV | 26 | RTVL-Ia | 224589806 | 40979271 | 906 |
| ENV | 27 | K-HERV | 224589807 | 10536437 | 222 |
| ENV | 28 | RTVL-Ia | 224589807 | 34231965 | 1263 |
| ENV | 29 | HERV-E | 224589807 | 72106212 | 333 |
| ENV | 30 | RTVL-Ia | 224589808 | 26558819 | 1284 |
| ENV | 31 | RTVL-Ia | 224589808 | 75164629 | 225 |
| ENV | 32 | RTVL-Ia | 224589809 | 41088888 | 198 |
| ENV | 33 | RTVL-Ia | 224589809 | 44456395 | 180 |
| ENV | 34 | RTVL-Ia | 224589810 | 11996337 | 201 |
| ENV | 35 | HERV-E | 224589810 | 20010094 | 174 |
| ENV | 36 | K-HERV | 224589810 | 20930917 | 1284 |
| ENV | 37 | HERV-K10 | 224589810 | 22762530 | 1107 |
| ENV | 38 | HERV-K10 | 224589810 | 23721803 | 795 |
| ENV | 39 | HERV-K10 | 224589810 | 28129450 | 1263 |
| ENV | 40 | MMTV | 224589810 | 36063297 | 1017 |
| ENV | 41 | RTVL-Ia | 224589810 | 52483588 | 540 |
| ENV | 42 | RTVL-Ia | 224589810 | 53172676 | 258 |
| ENV | 43 | HERV-K10 | 224589812 | 47898460 | 195 |
| ENV | 44 | K-HERV | 224589813 | 19934352 | 717 |
| ENV | 45 | RTVL-Ia | 224589814 | 18933178 | 1263 |
| ENV | 46 | RTVL-Ia | 224589814 | 22812684 | 270 |
| ENV | 47 | RTVL-Ia | 224589814 | 42501252 | 243 |
| ENV | 48 | K-HERV | 224589815 | 1933483 | 300 |
| ENV | 49 | RTVL-Ia | 224589815 | 9890692 | 1293 |
| ENV | 50 | HERV-E | 224589815 | 14081270 | 213 |
| ENV | 51 | K-HERV | 224589815 | 74878232 | 1440 |
| ENV | 52 | RTVL-Ia | 224589815 | 75606975 | 1188 |
| ENV | 53 | HERV-E | 224589815 | 87656965 | 306 |
| ENV | 54 | HERV-K10 | 224589815 | 87783703 | 1044 |
| ENV | 55 | HERV-K10 | 224589816 | 3980231 | 1011 |
| ENV | 56 | HERV-K10 | 224589816 | 4045256 | 630 |
| ENV | 57 | HERV-K10 | 224589816 | 9131379 | 735 |
| ENV | 58 | RTVL-Ia | 224589816 | 9667002 | 1011 |
| ENV | 59 | RTVL-Ia | 224589816 | 42321620 | 261 |
| ENV | 60 | HERV-K10 | 224589816 | 99935359 | 231 |
| ENV | 61 | HERV-E | 224589817 | 30487677 | 1263 |
| ENV | 62 | RTVL-Ia | 224589817 | 81564365 | 1284 |
| ENV | 63 | RTVL-Ia | 224589818 | 31297132 | 243 |
| ENV | 64 | RTVL-Ia | 224589818 | 35529505 | 234 |
| ENV | 65 | HERV-K10 | 224589818 | 66872395 | 192 |
| ENV | 66 | K-HERV | 224589818 | 78427579 | 2094 |
| ENV | 67 | HERV-K10 | 224589819 | 4622982 | 1263 |
| ENV | 68 | RTVL-Ia | 224589819 | 4631486 | 1263 |
| ENV | 69 | RTVL-Ia | 224589819 | 43858596 | 249 |
| ENV | 70 | HERV-K10 | 224589819 | 43862514 | 246 |
| ENV | 71 | RTVL-Ia | 224589819 | 65470824 | 765 |
| ENV | 72 | HERV-K10 | 224589819 | 70314099 | 207 |
| ENV | 73 | HERV-K10 | 224589820 | 7356322 | 1263 |
| ENV | 74 | HERV-K10 | 224589820 | 8062506 | 735 |
| ENV | 75 | HERV-K10 | 224589820 | 12074951 | 744 |
| ENV | 76 | K-HERV | 224589820 | 12317473 | 735 |
| ENV | 77 | HERV-E | 224589820 | 47176633 | 1182 |
| ENV | 78 | RTVL-Ia | 224589820 | 56626581 | 1326 |
| ENV | 79 | HERV-E | 224589820 | 80035781 | 270 |
| ENV | 80 | RTVL-Ia | 224589821 | 29382787 | 1368 |
| ENV | 81 | HERV-K10 | 224589821 | 90653107 | 261 |
| ENV | 82 | RTVL-Ia | 224589822 | 61960909 | 759 |
| ENV | 83 | K-HERV | 224589823 | 6825896 | 261 |
| ENV | 84 | RTVL-Ia | 224589823 | 6827412 | 1125 |
| ENV | 85 | RTVL-Ia | 224589823 | 7046287 | 174 |
| ENV | 86 | RTVL-Ia | 224589823 | 14581657 | 261 |
| ENV | 87 | RTVL-la | 224589823 | 21613337 | 192 |
| ENV | 88 | HERV-K10 | 224589823 | 23746172 | 768 |

^1^ U numbers, for clarity, derived from the Gypsy 2.0 database

^2^ HERV annotations as described in the Gypsy 2.0 database

^3^ GenBank/NCBI identifier number and start site
