# Supplementary material for: Analysis of Human Endogenous Retrovirus Expression in Multiple Sclerosis Plaques
Source: J Emerg Dis Virol. Author manuscript; Available in PMC 2017 Sep 1. (PMC5580941; doi:10.16966/2473-1846.133)
Supplement: qPCR std [file NIHMS900509-supplement-qPCR_std.pptx]

## Slide 1
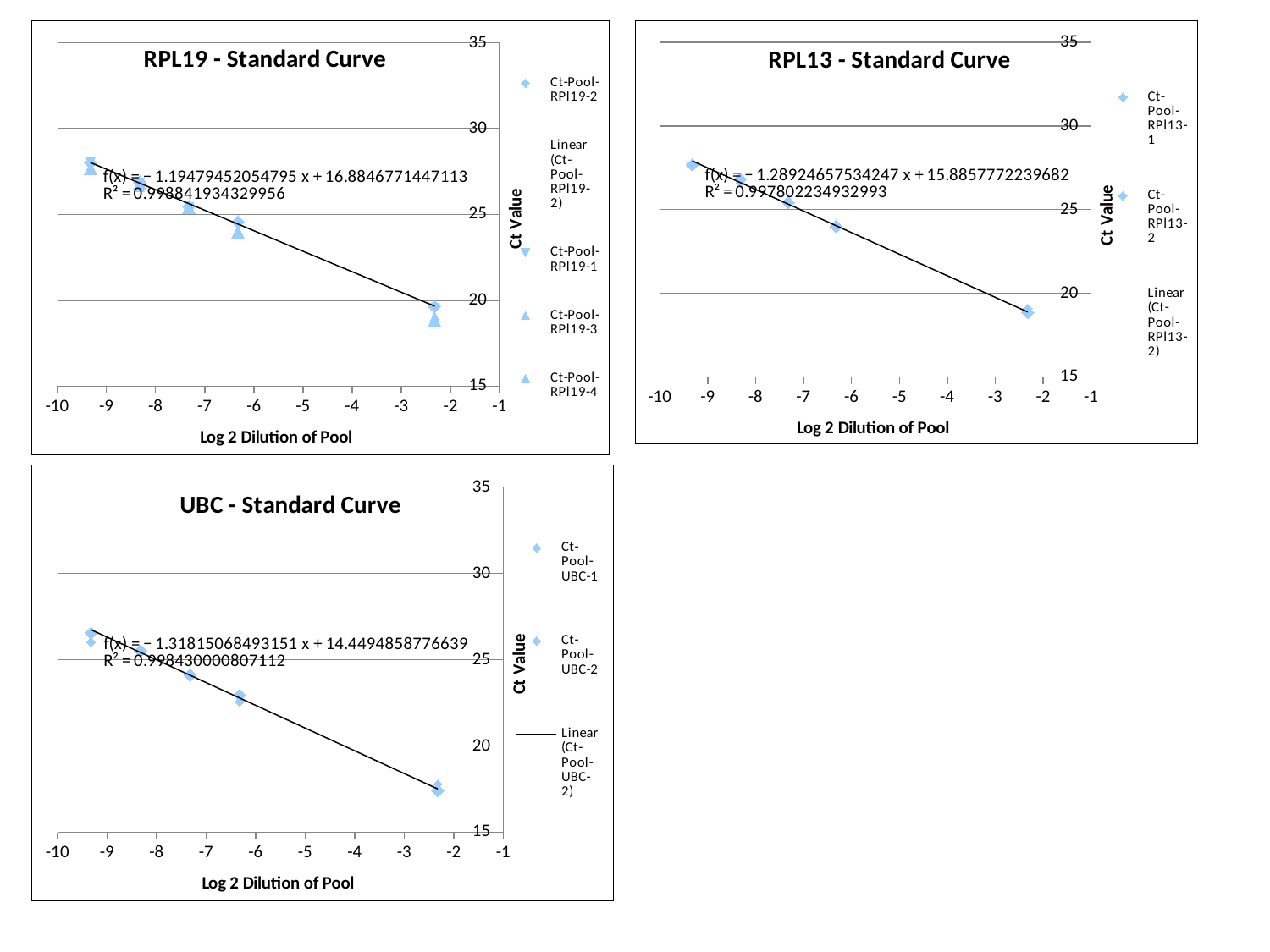

### Chart: RPL19 - Standard Curve
| Category | Ct-Pool-RPl19-2 | Ct-Pool-RPl19-1 | Ct-Pool-RPl19-3 | Ct-Pool-RPl19-4 |
|---|---|---|---|---|
### Chart: RPL13 - Standard Curve
| Category | Ct-Pool-RPl13-1 | Ct-Pool-RPl13-2 |
|---|---|---|
### Chart: UBC - Standard Curve
| Category | Ct-Pool-UBC-1 | Ct-Pool-UBC-2 |
|---|---|---|

## Slide 2
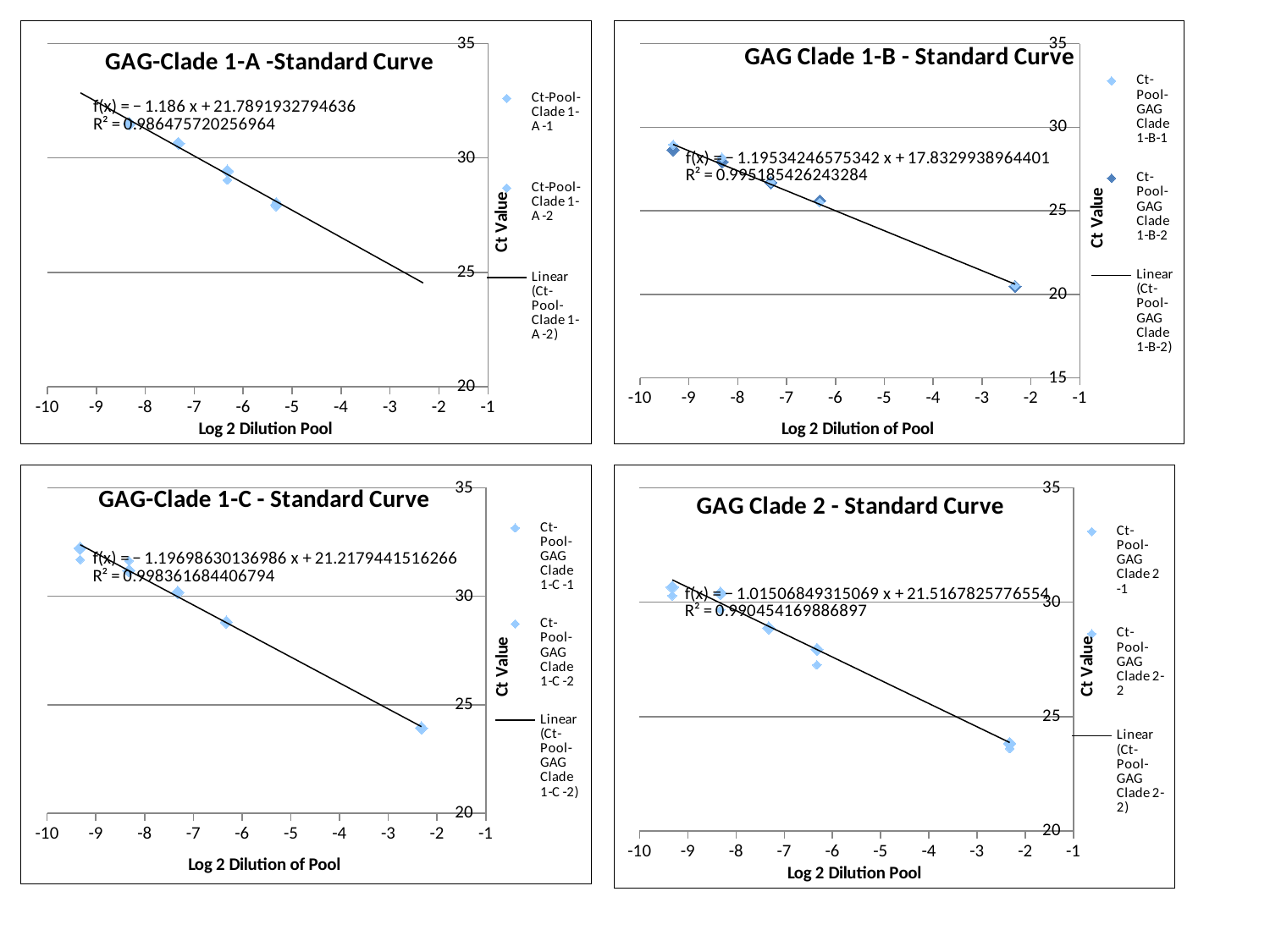

### Chart: GAG-Clade 1-A -Standard Curve
| Category | Ct-Pool-Clade 1-A -1 | Ct-Pool-Clade 1-A -2 |
|---|---|---|
### Chart: GAG Clade 1-B - Standard Curve
| Category | Ct-Pool-GAG Clade 1-B-1 | Ct-Pool-GAG Clade 1-B-2 |
|---|---|---|
### Chart: GAG-Clade 1-C - Standard Curve
| Category | Ct-Pool-GAG Clade 1-C -1 | Ct-Pool-GAG Clade 1-C -2 |
|---|---|---|
### Chart: GAG Clade 2 - Standard Curve
| Category | Ct-Pool-GAG Clade 2 -1 | Ct-Pool-GAG Clade 2-2 |
|---|---|---|

## Slide 3
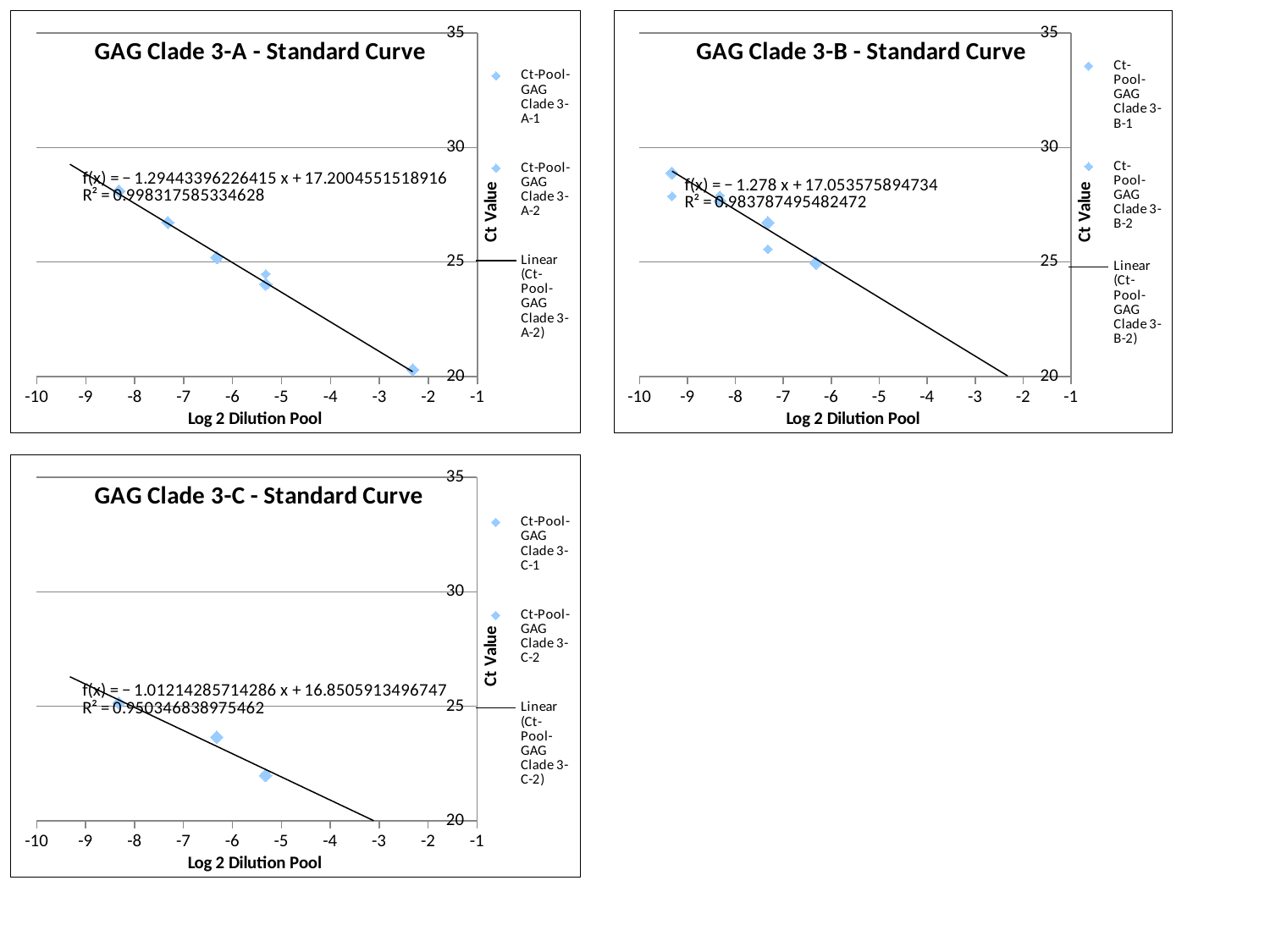

### Chart: GAG Clade 3-A - Standard Curve
| Category | Ct-Pool-GAG Clade 3-A-1 | Ct-Pool-GAG Clade 3-A-2 |
|---|---|---|
### Chart: GAG Clade 3-B - Standard Curve
| Category | Ct-Pool-GAG Clade 3-B-1 | Ct-Pool-GAG Clade 3-B-2 |
|---|---|---|
### Chart: GAG Clade 3-C - Standard Curve
| Category | Ct-Pool-GAG Clade 3-C-1 | Ct-Pool-GAG Clade 3-C-2 |
|---|---|---|

## Slide 4
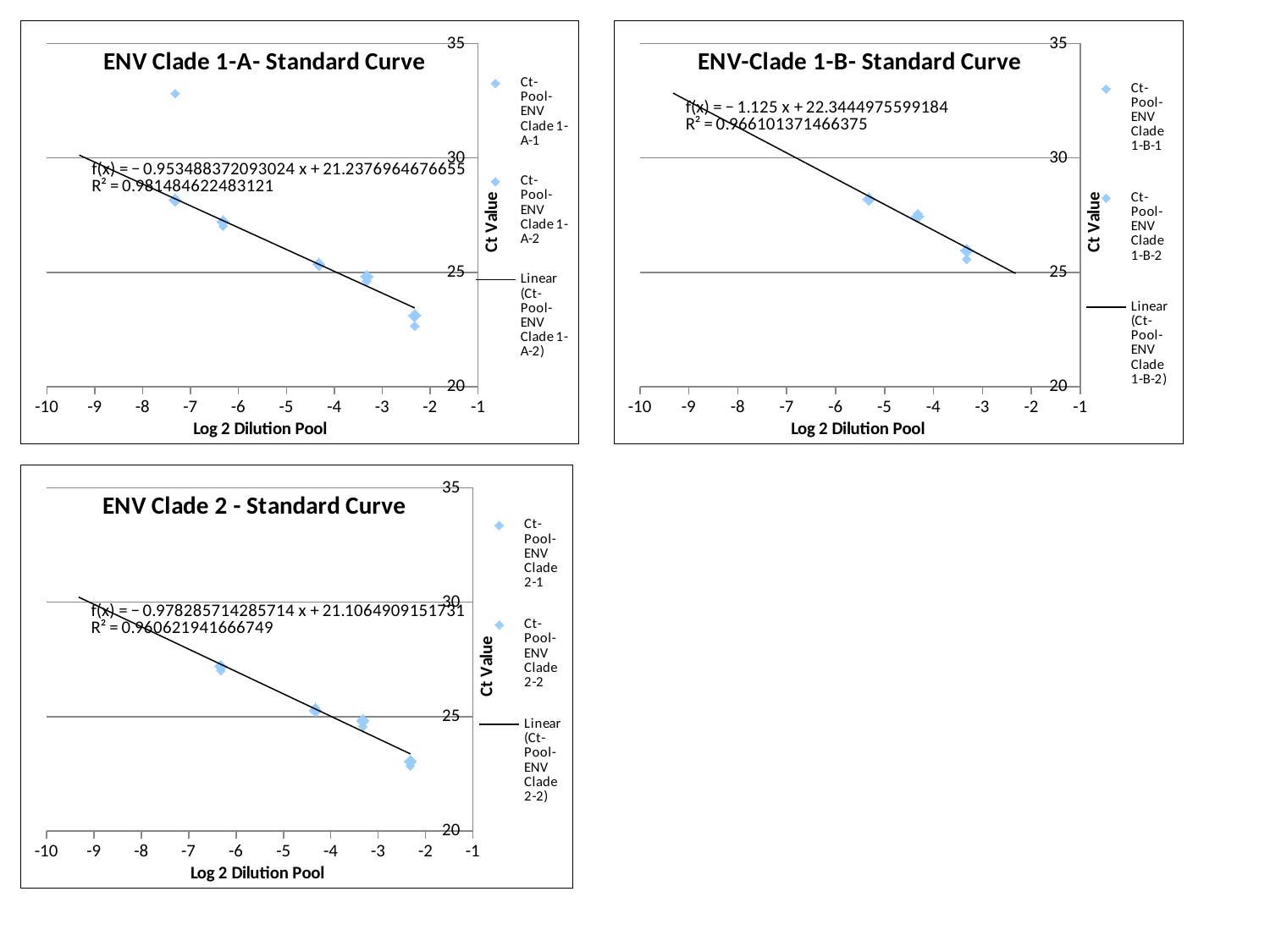

### Chart: ENV Clade 1-A- Standard Curve
| Category | Ct-Pool-ENV Clade 1-A-1 | Ct-Pool-ENV Clade 1-A-2 |
|---|---|---|
### Chart: ENV-Clade 1-B- Standard Curve
| Category | Ct-Pool-ENV Clade 1-B-1 | Ct-Pool-ENV Clade 1-B-2 |
|---|---|---|
### Chart: ENV Clade 2 - Standard Curve
| Category | Ct-Pool-ENV Clade 2-1 | Ct-Pool-ENV Clade 2-2 |
|---|---|---|

## Slide 5
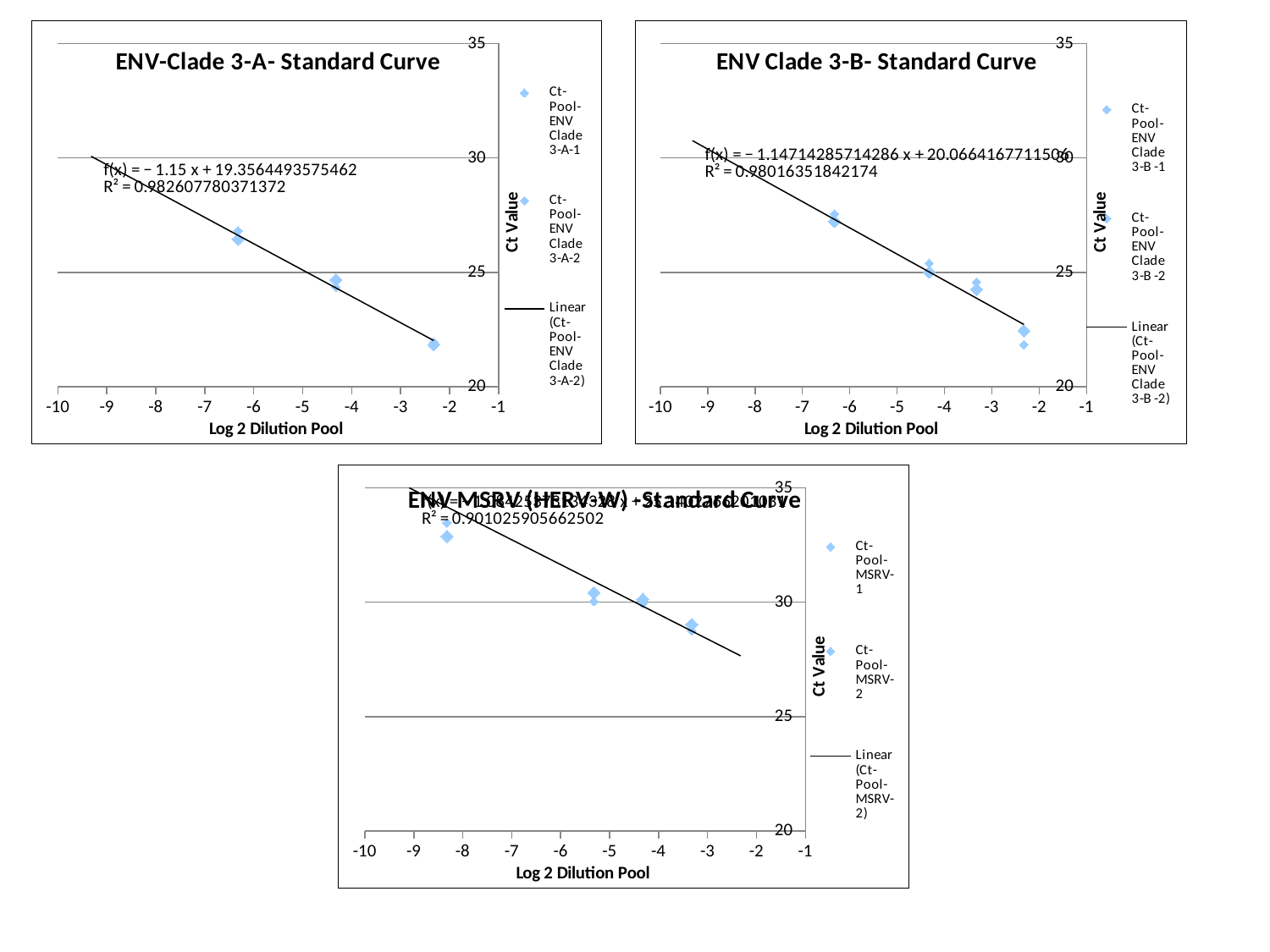

### Chart: ENV-Clade 3-A- Standard Curve
| Category | Ct-Pool-ENV Clade 3-A-1 | Ct-Pool-ENV Clade 3-A-2 |
|---|---|---|
### Chart: ENV Clade 3-B- Standard Curve
| Category | Ct-Pool-ENV Clade 3-B -1 | Ct-Pool-ENV Clade 3-B -2 |
|---|---|---|
### Chart: ENV MSRV (HERV-W) -Standard Curve
| Category | Ct-Pool-MSRV-1 | Ct-Pool-MSRV-2 |
|---|---|---|

## Slide 6
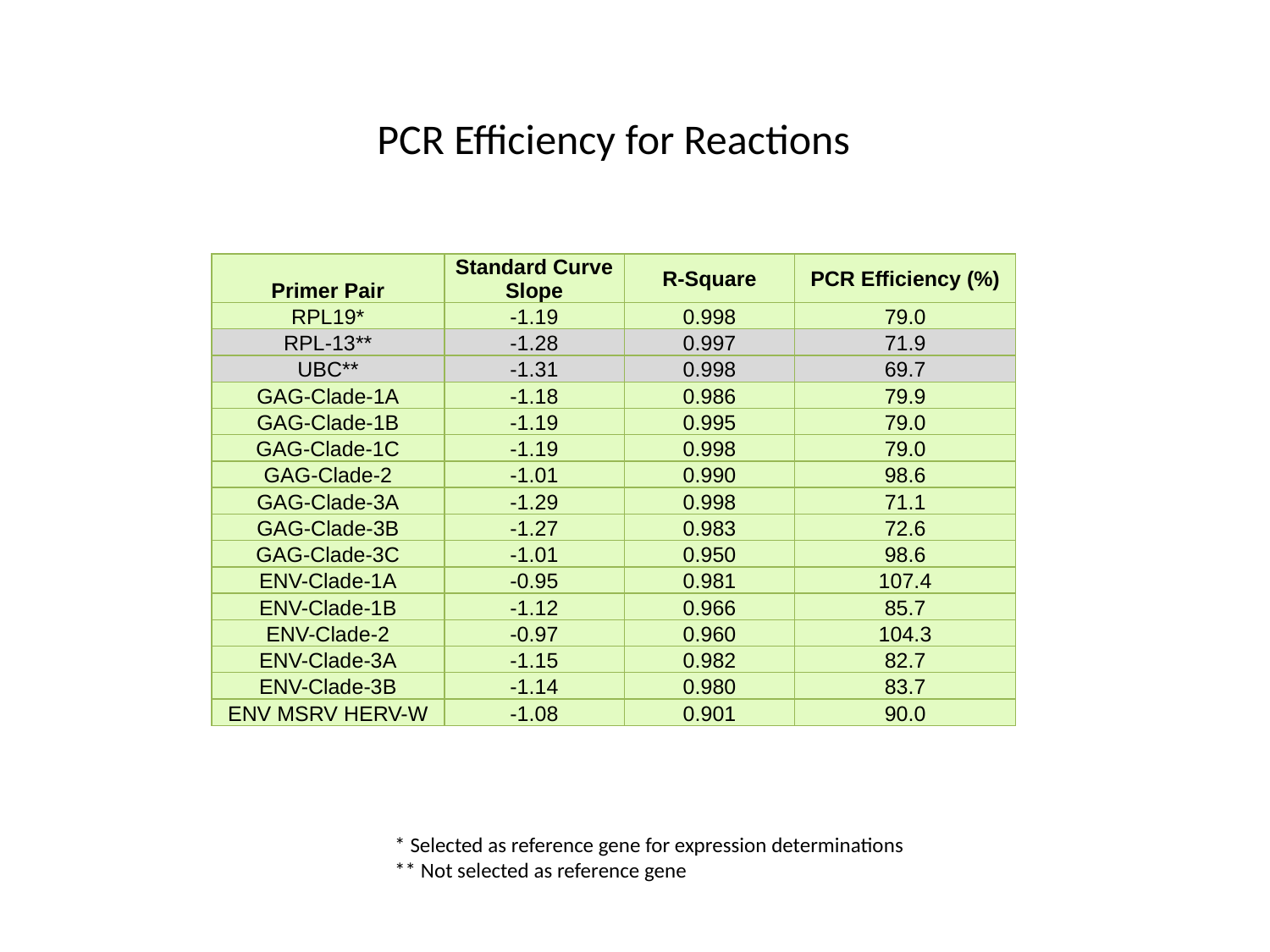

PCR Efficiency for Reactions
| Primer Pair | Standard Curve Slope | R-Square | PCR Efficiency (%) |
| --- | --- | --- | --- |
| RPL19\* | -1.19 | 0.998 | 79.0 |
| RPL-13\*\* | -1.28 | 0.997 | 71.9 |
| UBC\*\* | -1.31 | 0.998 | 69.7 |
| GAG-Clade-1A | -1.18 | 0.986 | 79.9 |
| GAG-Clade-1B | -1.19 | 0.995 | 79.0 |
| GAG-Clade-1C | -1.19 | 0.998 | 79.0 |
| GAG-Clade-2 | -1.01 | 0.990 | 98.6 |
| GAG-Clade-3A | -1.29 | 0.998 | 71.1 |
| GAG-Clade-3B | -1.27 | 0.983 | 72.6 |
| GAG-Clade-3C | -1.01 | 0.950 | 98.6 |
| ENV-Clade-1A | -0.95 | 0.981 | 107.4 |
| ENV-Clade-1B | -1.12 | 0.966 | 85.7 |
| ENV-Clade-2 | -0.97 | 0.960 | 104.3 |
| ENV-Clade-3A | -1.15 | 0.982 | 82.7 |
| ENV-Clade-3B | -1.14 | 0.980 | 83.7 |
| ENV MSRV HERV-W | -1.08 | 0.901 | 90.0 |
* Selected as reference gene for expression determinations
** Not selected as reference gene

## Slide 7
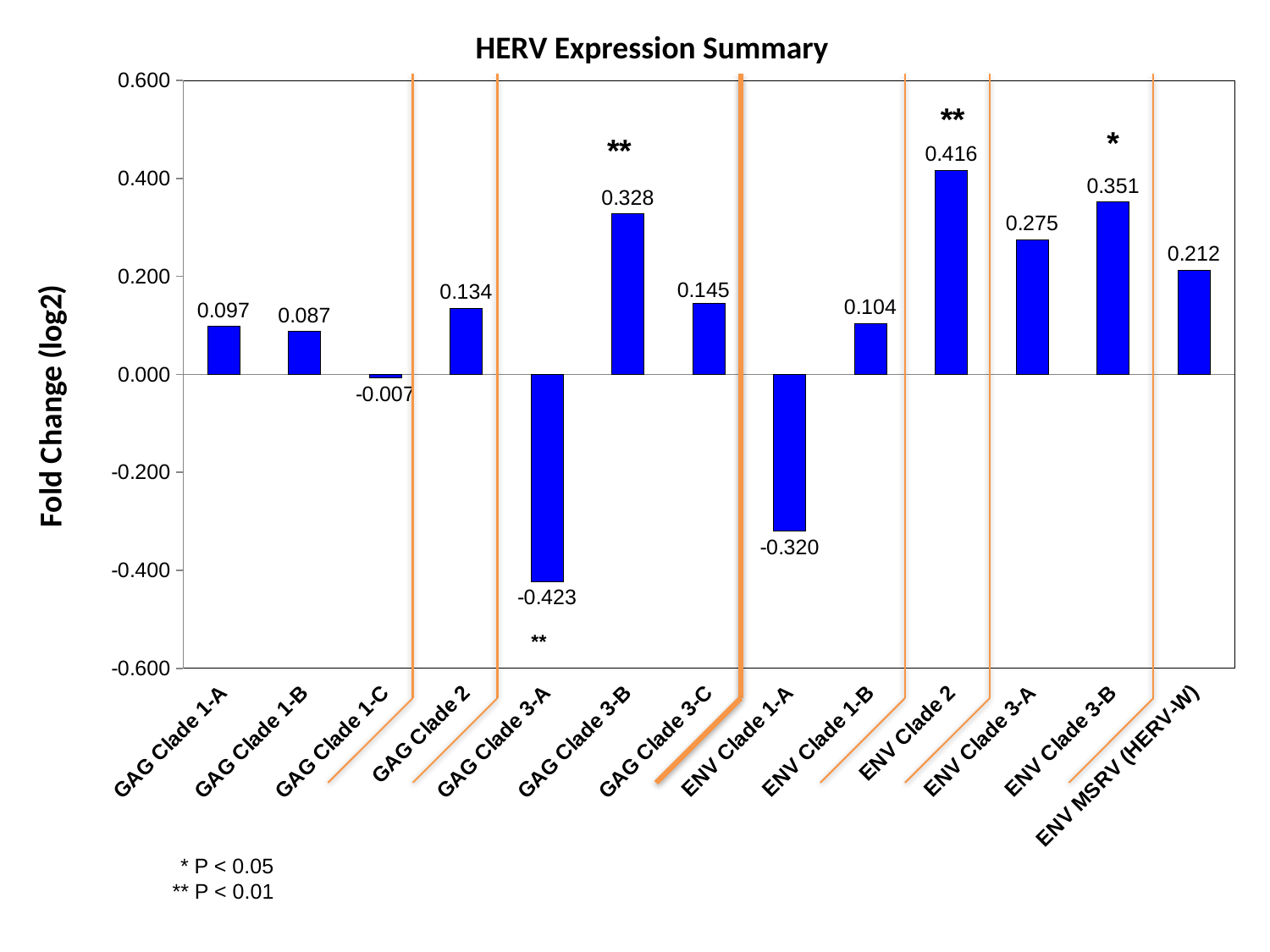

HERV Expression Summary
### Chart
| Category | Column1 |
|---|---|
| GAG Clade 1-A | 0.0974580263867288 |
| GAG Clade 1-B | 0.0873697933764583 |
| GAG Clade 1-C | -0.00699439330696891 |
| GAG Clade 2 | 0.13443596972692 |
| GAG Clade 3-A | -0.422919623288573 |
| GAG Clade 3-B | 0.327748058114968 |
| GAG Clade 3-C | 0.14501565282317 |
| ENV Clade 1-A | -0.320281015088766 |
| ENV Clade 1-B | 0.104277106032394 |
| ENV Clade 2 | 0.416484564012077 |
| ENV Clade 3-A | 0.274738547358958 |
| ENV Clade 3-B | 0.35136069104773 |
| ENV MSRV (HERV-W) | 0.212466247307635 |*
* P < 0.05
** P < 0.01

## Slide 8
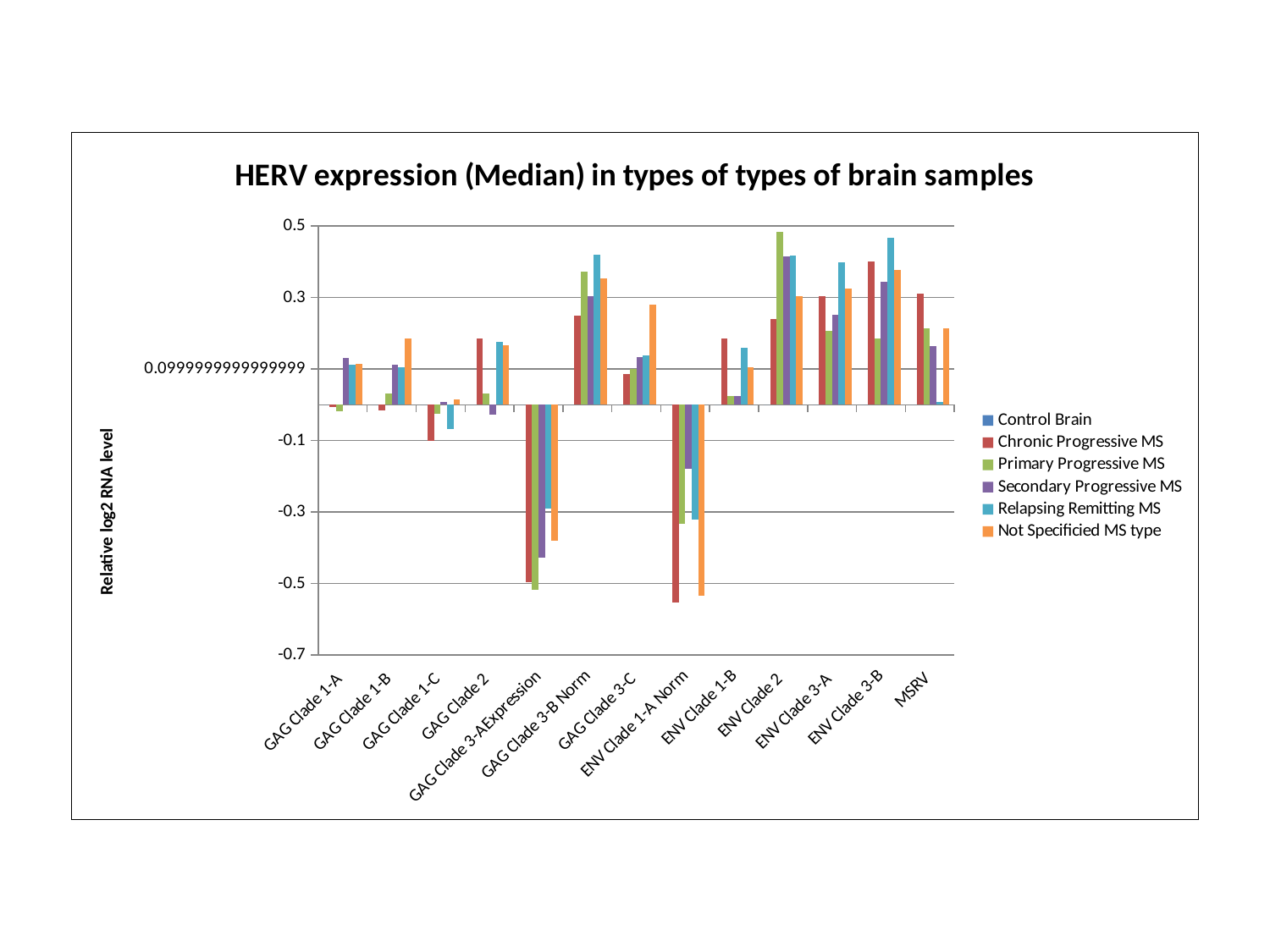

### Chart: HERV expression (Median) in types of types of brain samples
| Category | Control Brain | Chronic Progressive MS | Primary Progressive MS | Secondary Progressive MS | Relapsing Remitting MS | Not Specificied MS type |
|---|---|---|---|---|---|---|
| GAG Clade 1-A | 0.0 | -0.006194189395241811 | -0.01885861810536883 | 0.13096006716216213 | 0.11120320869438688 | 0.11477443607461182 |
| GAG Clade 1-B | 0.0 | -0.01668478975067242 | 0.03249007581741818 | 0.1107589867933057 | 0.10578311705885637 | 0.18481491033541306 |
| GAG Clade 1-C | 0.0 | -0.1017325636287262 | -0.024513616518653455 | 0.007347502646947321 | -0.06729066152335078 | 0.014784146260137049 |
| GAG Clade 2 | 0.0 | 0.18479965652220856 | 0.03127494703042018 | -0.02723116361140905 | 0.1761314884473028 | 0.16572218681654208 |
| GAG Clade 3-AExpression | 0.0 | -0.49544074654470605 | -0.5183460294185642 | -0.42692274713920675 | -0.29155018043854686 | -0.3812513448732082 |
| GAG Clade 3-B Norm | 0.0 | 0.24839905575791443 | 0.37277727852627196 | 0.30452025607739375 | 0.4190726296519748 | 0.35429059035490584 |
| GAG Clade 3-C | 0.0 | 0.0865692988898214 | 0.10066996890090324 | 0.13313812089676513 | 0.13868496136004363 | 0.28090478351766396 |
| ENV Clade 1-A Norm | 0.0 | -0.5523057668389413 | -0.3334683954407732 | -0.1784469584191718 | -0.3202810150887656 | -0.5340544011921794 |
| ENV Clade 1-B | 0.0 | 0.185727071461407 | 0.02343168533835624 | 0.023747202500005143 | 0.15808500730337077 | 0.10593545417885558 |
| ENV Clade 2 | 0.0 | 0.24036555983166685 | 0.484288384080686 | 0.41539208418744483 | 0.41648456401207734 | 0.3030492981817075 |
| ENV Clade 3-A | 0.0 | 0.30367362751922256 | 0.2060978077625438 | 0.25133533866769636 | 0.3989854915056079 | 0.3253764099425783 |
| ENV Clade 3-B | 0.0 | 0.39968184090812453 | 0.18570822574896328 | 0.34420120858132097 | 0.4658386445240466 | 0.37619882731520204 |
| MSRV | 0.0 | 0.3106667453908328 | 0.2133625547525418 | 0.16297965249172747 | 0.007182041085218582 | 0.2124662473076352 |
